# Serine phosphorylation of CesT by the type III secretion system effectors NleH1 and NleH2 regulates antagonization of CsrA in enteropathogenic *Escherichia coli*

**DOI:** 10.1128/iai.00027-26

**Published:** 2026-02-26

**Authors:** Esther Tang, Angeline C. Beltran, Senthuran Mahendradeva, Abiali A. Badani, Dustin J. Little

**Affiliations:** 1 Department of Chemistry and Biology, Toronto Metropolitan University 468134, Toronto, Ontario, Canada; University of California Davis, Davis, California, USA

**Keywords:** secretion systems, protein phosphorylation, effector functions, *Escherichia coli*, protein chaperone, host-pathogen interactions, posttranscriptional RNA-binding proteins, protein translocation, serine/threonine kinases

## Abstract

Enteropathogenic *Escherichia coli* (EPEC) utilizes the type 3 secretion system (T3SS) to translocate effector proteins into host cells that hijack cell signaling pathways to promote infection, colonization, and survival. Prior studies have shown that phosphorylation of Y152 and Y153 on the multi-effector T3SS chaperone CesT influences effector secretion and host colonization dynamics. However, additional phosphosites, the kinase(s) responsible for CesT phosphorylation, and the downstream effect(s) remain poorly characterized. Herein, we used a combination of biochemical and functional assays to show CesT tyrosine phosphorylation levels do not change in the absence or presence of the T3SS effector kinases NleH1 and/or NleH2. Instead, we demonstrate that when NleH1 and NleH2 are in complex with CesT, they function as serine kinases that target the S146 and/or S147 phosphosites, consistent with their role as Ser/Thr kinases on host nuclear factor-kappa B signaling pathways. We show that the CesT S146E, S147A, and S147E variants have an impaired ability to interact with CsrA using pull-down assays, and an EPEC Δ*nleH1* Δ*nleH2* strain displays increased NleA translation and host translocation compared to wild-type EPEC, supporting the role of CesT S146 and S147 phosphorylation in regulating CsrA repression of *nleA* mRNA. Our data support a model where the phosphorylation of CesT at S146 and/or S147 influences NleA translation levels by impairing CesT-CsrA complex formation, which in turn modulates the levels of NleA translation and translocation into host cells. This study further highlights the complex post-translational regulatory functioning of CesT in orchestrating T3SS effector secretion hierarchy during infection.

## INTRODUCTION

Enteropathogenic and enterohemorrhagic *Escherichia coli* (EPEC and EHEC, respectively) are human pathogens that cause gastrointestinal diseases (1). EPEC and EHEC can be distinguished from other *E. coli* pathotypes by the presence of the 35.6 kilobase chromosomal pathogenicity island known as the Locus of Enterocyte Effacement (LEE) (1–4). The expression of LEE genes confers the development of attaching and effacing (A/E) lesions on intestinal epithelia during infection (5). A/E lesion formation is dependent on the function of the type 3 secretion system (T3SS). The T3SS is a self-produced injectisome that serves as a protein conduit to secrete effector proteins into host cells, which modulate host cell signaling pathways to promote host actin polymerization and bacterial colonization (1, 6). Not surprisingly, the T3SS is a key virulence determinant in a wide range of gram-negative pathogens, such as EPEC, EHEC, *Salmonella* spp., *Shigella* spp., *Chlamydia* spp., *Yersinia* spp., *Citrobacter rodentium, Bordetella pertussis,* and *Pseudomonas aeruginosa* (among others) (6–10). However, the arsenal of effectors and their respective host targets varies widely between pathogens.

Effector secretion in EPEC has been shown to follow a hierarchical process, with the translocated intimin receptor (Tir) as the first effector translocated into host cells. Tir secretion then triggers the release of additional effectors, as a *tir* deletion has been shown to be impaired for the secretion of at least six different effectors into culture supernatants or directly into host cells (11, 12). Moreover, Tir secretion is dependent on the function of the T3SS chaperone, CesT (11–13). CesT regulates the secretion of Tir and at least eight other effectors, while the only other T3SS chaperone in EPEC, CesF, regulates EspF secretion (12, 14). Although CesT is a multi-cargo chaperone, it has been implicated in effector secretion hierarchy through multiple mechanisms. One mechanism is through its physical interaction with effectors. CesT has been shown to bind to a conserved N-terminal region on most effectors just downstream of the secretion signal (11). However, CesT was shown to bind to the N-terminus and C-terminus of Tir, a mechanism that plays a role in preferential Tir secretion during infection (15). CesT has also been shown to post-transcriptionally regulate NleA translation and secretion by binding to the mRNA regulatory protein CsrA (16). CsrA was found to bind to the 5′ untranslated region of the *nleA* mRNA, where it forms a clamp-like structure that sequesters the ribosomal binding site, inhibiting ribosome attachment and subsequent translation (16). To derepress *nleA* and allow for NleA translation and secretion, CesT can sequester CsrA to displace it from the *nleA* mRNA, but this only occurs in the host-attached state when the effector pool has been depleted and free cytosolic CesT is increased (16). Moreover, the C-terminus of CesT has also been implicated in effector secretion hierarchy in EHEC and EPEC. Hansen et al. found in an EHEC phosphoproteome study that Y152 and Y153 of CesT can be phosphorylated (17). Interestingly, Ye et al. determined the CsrA-CesT crystal structure and showed that CesT Y152 makes a critical interaction with CsrA, and the Y152A variant abolished their interaction (18). Moreover, this was further supported by Runte et al. in EPEC, where the CesT Y152F variant was impaired for NleA translocation into host cells (19), and a CesT Y152F Y153F chromosomal variant showed reduced colonization and faster clearing of *C. rodentium* from the intestines of mice in a murine model of infection (19).

The importance of the CesT C-terminus in effector secretion has also been reported by Ramu et al*.,* where they showed it was important in translocation efficiency of NleA but dispensable for Tir translocation (20). Additionally, they identified mutations to tandem serine residues S146 and S147 that led to a decrease in NleA secretion in EPEC (20). More recently, Yadav et al. provided evidence that the T3SS effector NleH2 in EPEC could phosphorylate CesT prior to effector translocation and proposed that NleH2 was this unknown kinase that preferentially targets the Y153 phosphosite based on mutagenesis studies in combination with ATPase assays (21). However, NleH2 has been previously shown to be a Ser/Thr kinase targeting host nuclear factor-kappa B signaling pathways (22, 23). Although there are cases of Ser/Thr kinases acting on Tyr residues, Ramu et al. showed that mutations to CesT residues S145, S146, and S147 at the C-terminal region had reduced effector secretion efficiency (20). Considering this, it is more plausible that NleH2 acts as a Ser/Thr kinase on the CesT C-terminus, and mutations to Y152 and/or Y153 could alter binding to NleH2, reducing kinase activity.

In this study, we show that NleH1 and NleH2 function as Ser kinases on CesT, but only when they form a stable complex. We identify S146 and S147 on CesT as the major phosphosites, and the S146E, S147A, and S147E variants negatively influence CesT interaction with CsrA. This suggests that phosphorylation of CesT at S146 and/or S147 can further regulate NleA translation, as an EPEC Δ*nleH1* Δ*nleH2* strain showed increased NleA levels and translocation into host cells. This study further supports that phosphorylation is a contributing mechanism for fine-tuning T3SS-dependent virulence factor delivery during infection.

## RESULTS

### CesT tyrosine phosphorylation is not altered in EPEC Δ*nleH1* and/or Δ*nleH2*

**Fig 1:**
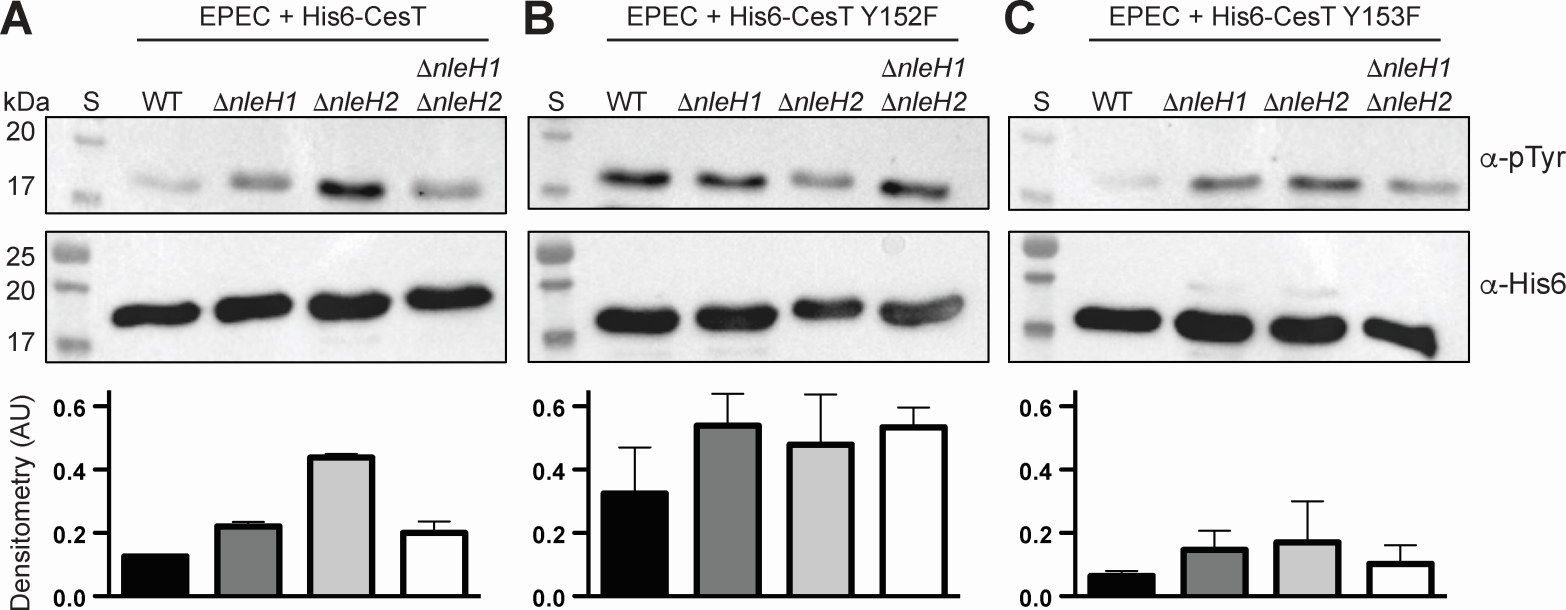
Tyrosine phosphorylation analysis of CesT, CesT Y152F, and CesT Y153F in EPEC WT, Δ*nleH1*, Δ*nleH2,* and Δ*nleH1* Δ*nleH2* genetic backgrounds. (**A**) His6-CesT, (**B**) His6-CesT Y152F, and (**C**) His6-CesT Y153F were expressed in *trans* from EPEC grown in M9 optimized media, and CesT or variants thereof were isolated using Ni-NTA affinity agarose resin after cellular lysis. Elution samples were analyzed via western blot using α-pTyr and α-His6 for tyrosine phosphorylation and CesT levels, respectively. Densitometry was calculated as the ratio of phosphorylated to total protein levels using Image Lab software. Bars represent the mean ± SEM from two replicate experiments. S, molecular weight standards.

A recent study suggested that NleH2 was a tyrosine kinase for CesT, specifically targeting the Y153 phosphosite on the CesT C-terminus (21). However, the biochemical assay performed to detect ADP generation was an indirect measure of kinase activity and did not rule out alternative explanations for those results. One such explanation could be that the CesT Y153F variant is a poor substrate for NleH2 kinase activity by altering its binding to the active site. Thus, we posited that if NleH1 or NleH2 function as tyrosine kinases for CesT, then deletion of *nleH1* and/or *nleH2* in EPEC would alter CesT tyrosine phosphorylation levels. To test this, we cloned and transformed a plasmid encoding His6-CesT into EPEC wild type (WT), Δ*nleH1,* Δ*nleH2,* and Δ*nleH1* Δ*nleH2*. We then expressed His6-CesT, or variants thereof, in each strain grown in M9 optimized medium that mimics T3SS-inducing conditions (24). His6-CesT and the phosphosite variants were captured and isolated using Ni-NTA affinity agarose and analyzed using α-pTyr and α-His6 western blots to determine whether CesT is phosphorylated under these specific conditions. Western blot analysis showed that knocking out *nleH1, nleH2*, or both *nleH1* and *nleH2* did not decrease tyrosine phosphorylation levels of CesT in EPEC but showed similar or greater tyrosine phosphorylation levels compared to WT, with Δ*nleH2* showing the highest levels (Fig. 1A). This strongly suggests that NleH1 and NleH2 do not play a major or significant role in tyrosine phosphorylation of CesT in EPEC. Next, we wanted to test if individual tyrosine phosphosites showed the same pattern. To do this, we conducted the same experiment but expressed the His6-CesT Y152F or His6-CesT Y153F mutants. The His6-CesT Y152F mutant showed a similar phosphotyrosine profile between EPEC WT, Δ*nleH1*, Δ*nleH2*, and Δ*nleH1* Δ*nleH2* strains (Fig. 1B). Likewise, the His6-CesT Y153F mutant also showed similar phosphotyrosine profiles between EPEC WT, Δ*nleH1*, Δ*nleH2*, and Δ*nleH1* Δ*nleH2* strains (Fig. 1C), albeit all strains showed reduced levels overall compared to the WT His6-CesT. Considering these results, they suggest that the mechanism of NleH1 and/or NleH2 phosphorylation of CesT is not through Y153, which was proposed previously by Yadav et al. (21).

### The NleH2 kinase domain alone does not phosphorylate CesT

**Fig 2:**
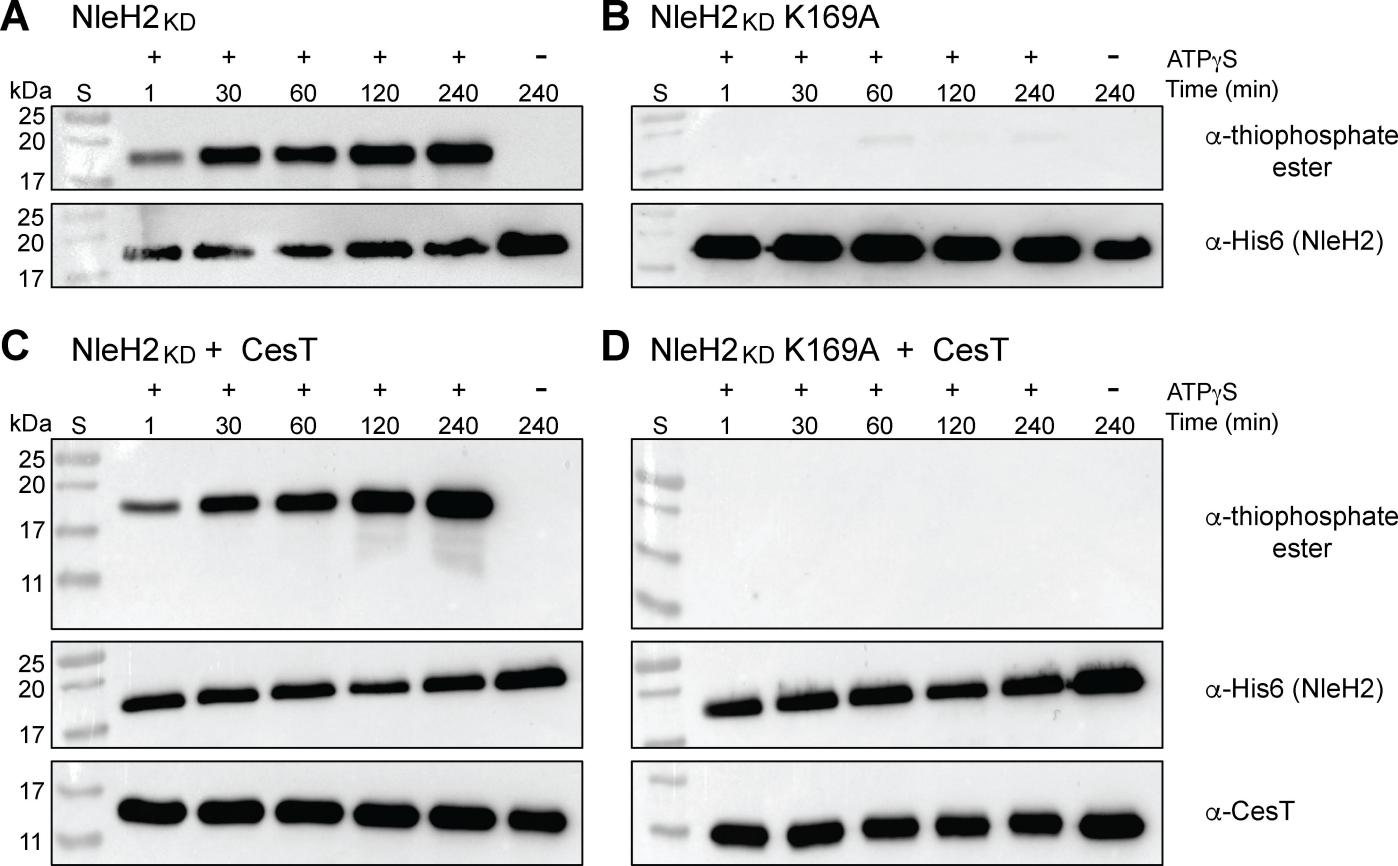
The NleH2 kinase domain autophosphorylates but does not phosphorylate CesT. Purified (**A**) NleH2_KD_, (**B**) NleH2_KD_ K169A, (**C**) NleH2_KD_ and CesT, and (**D**) NleH2_KD_ K169A and CesT (all at 4 µM) were incubated with 1 mM ATPγS and 2 mM MgCl_2_. Samples were taken at different time points and alkylated by incubating with 2.5 mM PNBM for 1 h. Samples were then run on SDS-PAGE and analyzed using α-thiophosphate ester, α-His6 (NleH2_KD_), and α-CesT western blot. S, molecular weight standards. The blots shown are representative of ≥2 independent experiments.

To gain a better understanding toward the phosphorylation activity of NleH2 and specificity for CesT, we developed a kinase assay that utilizes ATPγS for direct detection. ATPγS is an ATP analog that contains a thiol group at the γ-phosphate, which can be alkylated and then visualized using an α-thiophosphate ester antibody. This allows for direct tracking of phosphotransfer events by a kinase onto a substrate. To validate our kinase assay, we first tested the ability of the NleH2 kinase domain (NleH2_KD_), containing residues 140–303, and the NleH2_KD_ inactive mutant K169A to autophosphorylate in the presence of ATPγS. The α-thiophosphate ester western blot shows that isolated NleH2_KD_ can autophosphorylate in a time-dependent manner (Fig. 2A). Additionally, kinase activity is abolished in the NleH2_KD_ K169A inactive mutant, as the α-thiophosphate ester blot showed no detectable levels in the same time frame (Fig. 2B). Now that we established specificity in our assay, we wanted to determine if the NleH2_KD_ alone could phosphorylate CesT. When NleH2_KD_ was assayed under the same conditions but now with the addition of CesT to the mixture, NleH2 autophosphorylation was still visible, but no additional signal between 11 and 17 kDa, which corresponds to CesT, was observed in the α-thiophosphate ester blot (Fig. 2C). Moreover, assaying NleH2_KD_ K169A with CesT showed an inability for NleH2_KD_ to autophosphorylate or phosphorylate CesT (Fig. 2D). This suggests that NleH2_KD_ alone does not display phosphorylation activity on CesT.

### NleH1 and NleH2 can phosphorylate CesT when in complex

**Fig 3:**
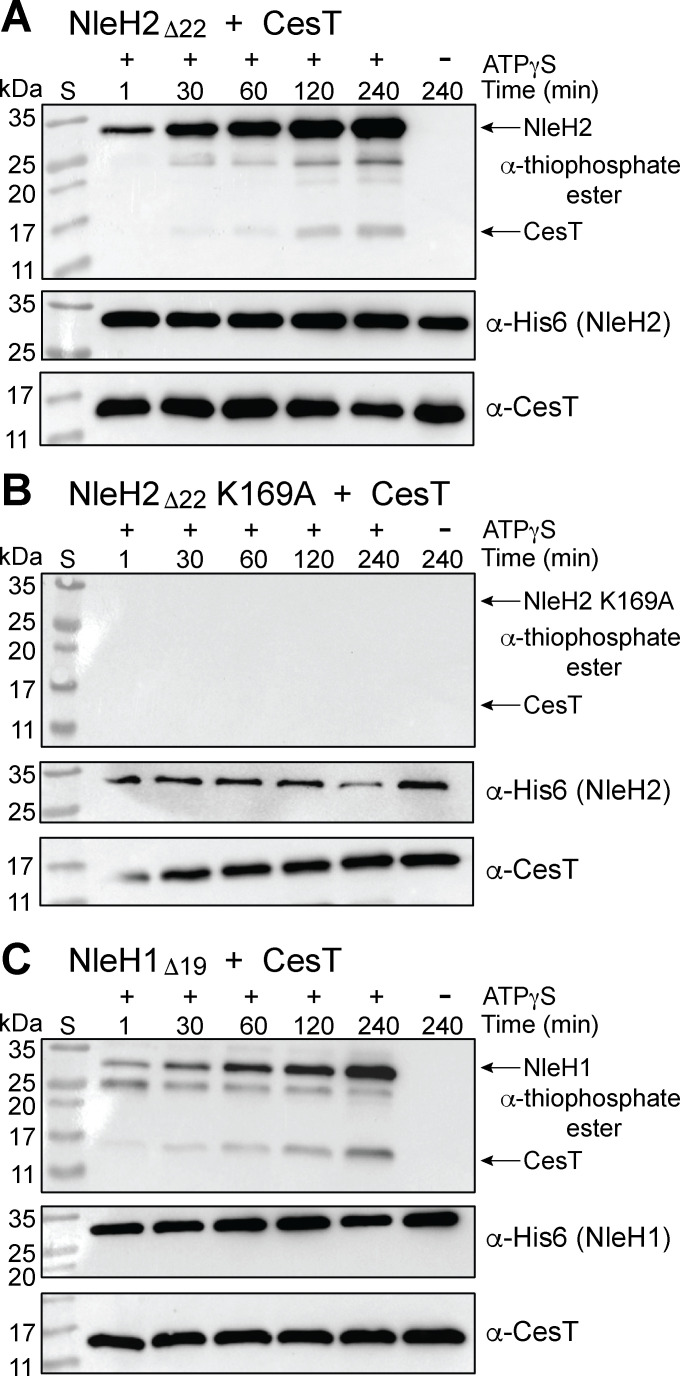
NleH1 and NleH2 can phosphorylate CesT when they form a complex. Purified (**A**) His6-NleH2_Δ22_, (**B**) His6-NleH2_Δ22_ K169A, and (**C**) His6-NleH1_Δ19_ were incubated with CesT (all at 4 µM), 1 mM ATPγS, and 2 mM MgCl_2_. Samples were taken at different time points and alkylated by incubating with 2.5 mM PNBM for 1 h. Samples were then run on SDS-PAGE and analyzed using α-thiophosphate ester, α-His6, and α-CesT western blot. The blots shown are representative of ≥2 independent experiments.

We hypothesized that a confounding factor of the NleH2_KD_ kinase assay results could be that NleH2_KD_ lacks the specific CesT interaction region (residues 25–97) recently determined (21). This, in turn, could prevent a productive interaction between NleH2_KD_ and CesT, limiting CesT phosphorylation (21). To test this, we performed the same kinase assay, but using His6-NleH2 23–303 (NleH2_Δ22_) co-expressed and purified with CesT. Under these conditions, NleH2 displayed autophosphorylation similar to NleH2_KD_ alone, as evident in the α-thiophosphate ester blot (Fig. 3A). However, we now observed an increase in phosphorylation levels over time in the α-thiophosphate ester blot for CesT at the ~17 kDa marker (Fig. 3A). Moreover, this phosphorylation activity was abolished when we conducted the kinase assay using the NleH2_Δ22_ K169A inactive mutant, indicating assay specificity (Fig. 3B). As NleH1 shares 84% sequence similarity with NleH2 in EPEC, we were also interested in testing if NleH1 could autophosphorylate and phosphorylate CesT using the same kinase assay. Thus, we co-expressed and purified His6-NleH1 20–293 (NleH1_Δ19_) and CesT and subjected it to the same assay conditions. Similar to NleH2_Δ22_, NleH1_Δ19_ was also able to autophosphorylate and phosphorylate CesT, as evident in the α-thiophosphate ester blot (Fig. 3C). Together, these results suggest that NleH1 and NleH2 function as kinases on CesT, but only when they form a stable complex.

### NleH2 shows impaired kinase activity on CesT S146A and S147A phosphomutants

**Fig 4:**
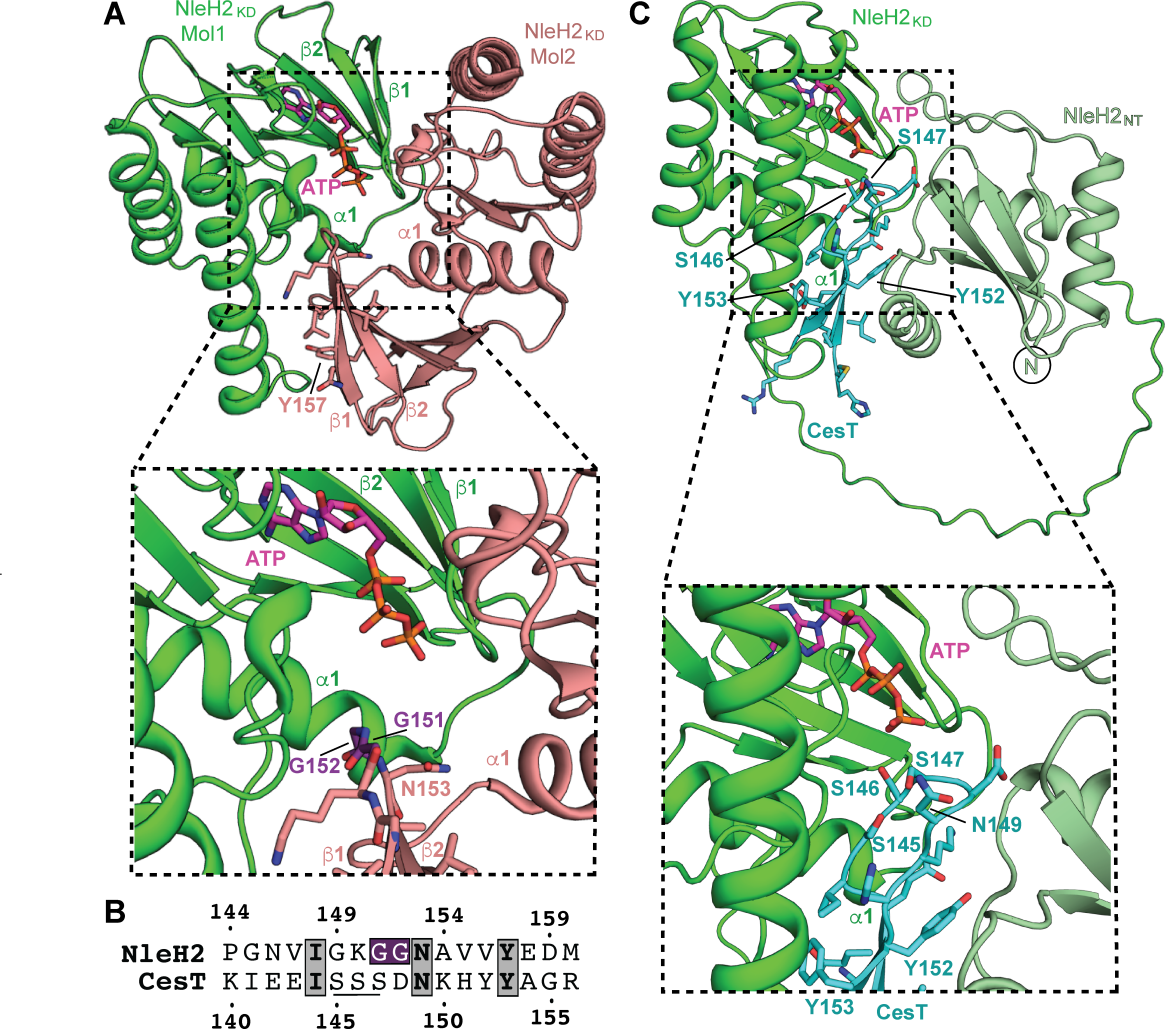
Structural modeling of NleH2_KD_ and NleH2 bound to CesT 138–156. (**A**) The structure of NleH2_KD_ (PDB 4LRK) shows a self-interacting dimer that is in close proximity to the proposed kinase active site. ATP (magenta) was modeled in for reference. (**B**) Sequence alignment of NleH2 144–160 and CesT 138–156 shows conserved residues and location of the triple serine phosphosite (underlined). (**C**) Model of the AlphaFold 3-predicted complex of the full-length NleH2 (kinase domain green, N-terminal domain smudge green) bound to CesT 138–156 (cyan), and ATP (magenta). Interactions between CesT S146 and/or S147 with the γ-phosphate of ATP in the NleH2 active site are approximate. Structures were visualized with PyMOL.

Now that we established that NleH1 and NleH2 act as kinases for CesT but do not alter tyrosine phosphorylation levels, we sought to identify the specific phosphosites. To do this, we expressed and purified His6-CesT and the non-tyrosine phosphorylatable His6-CesT Y152F Y153F mutant from EPEC cultured in T3SS-inducing medium. The CesT samples were then separated and isolated using Ni-NTA resin and analyzed using phosbind acrylamide SDS-PAGE. Under these conditions, phosbind acrylamide will alter the migration pattern of phosphoproteins within the SDS-PAGE gel. We observed that WT CesT displayed three major species (Fig. S1). Interestingly, the CesT Y152F Y153F variant also showed the same three major species, with no discernible difference in migration pattern to WT CesT. This strongly suggests that other major phosphosites exist beside the tandem Y152 and Y153 sites in CesT. To identify the phosphorylated residues in these samples, we excised the protein bands and submitted the samples for tryptic digest and liquid chromatography tandem mass spectrometry (LC-MS/MS), then analyzed the data for phosphopeptide identification using the Scaffold post-translational modification (PTM) software. This revealed multiple phosphorylation species on CesT (Table S4). Of particular interest was a tandem triple serine site, S145, S146, and S147. The data were not robust enough (lower Ascore) to definitively discern if S145, S146, or S147 was the specific serine phosphorylated in the peptide with 100% confidence, but in each case, the probability that one of those three serine residues was phosphorylated ranged between 97 and 100%. Interestingly, this triple serine phosphosite was also identified by Yadav et al. when CesT was co-purified with NleH2 (residues 25–303) and assayed for kinase activity (21). Thus, these sites were selected for further investigation as they are in close proximity to the Y152 and Y153 phosphosites, and these serine residues have previously been shown to be important for effector secretion (20). To probe this further, we investigated the sequence and structure of CesT residues 138–156 as a possible substrate for NleH2. The previously determined crystal structure of NleH2_KD_ (PDB 4LRK) showed a central dimeric arrangement of two protomers in the asymmetric unit that pack in a manner representative of a possible substrate-binding cleft (Fig. 4A) (25). As NleH2_KD_ crystallized in the absence of ATP, we modeled ATP into the active site to further support our analysis. In this snapshot, the γ-phosphate of ATP is within 5–6 Å of G151 and G152 that forms part of the loop between β1 and β2 of the second NleH2_KD_ protomer (Fig. 4A). Interestingly, if you align this self-bound NleH2 sequence with that of the CesT C-terminus (residues 138–156), several key residues overlap in the binding site such as CesT Y153 and NleH2 Y157, and the triple serine site of CesT with G151 and G152 of NleH2 (Fig. 4B). Thus, to get a better approximation of how the CesT C-terminus might bind to the kinase active site of NleH2, we performed AlphaFold modeling using NleH2_KD_, ATP, and a CesT peptide containing residues 138–156 (Fig. 4C). Here, we can see that the CesT C-terminal peptide mirrors a very similar binding mode as the other NleH2_KD_ protomer from Fig. 4A. CesT 138–156 adopts a β-hairpin structure, where the hairpin loop contains S145, S146, and S147 that are approximately 6.4, 3.0, and 2.4 Å away from the γ-phosphate of ATP. This would place S146 and S147 in a productive position to act as a nucleophile for phosphotransfer from NleH2. Moreover, we also observed other important interactions from our predictions along the binding cleft. Notably, Y152 and Y153 independently bind into small pockets that help bridge the NleH2_KD_ α1 and the NleH2 N-terminal domain (Fig. 4C). Together, these data further support that S145, S146, and/or S147 are the likely CesT phosphosites for NleH2, whereas Y152 and Y153 play a role in binding.

**Fig 5:**
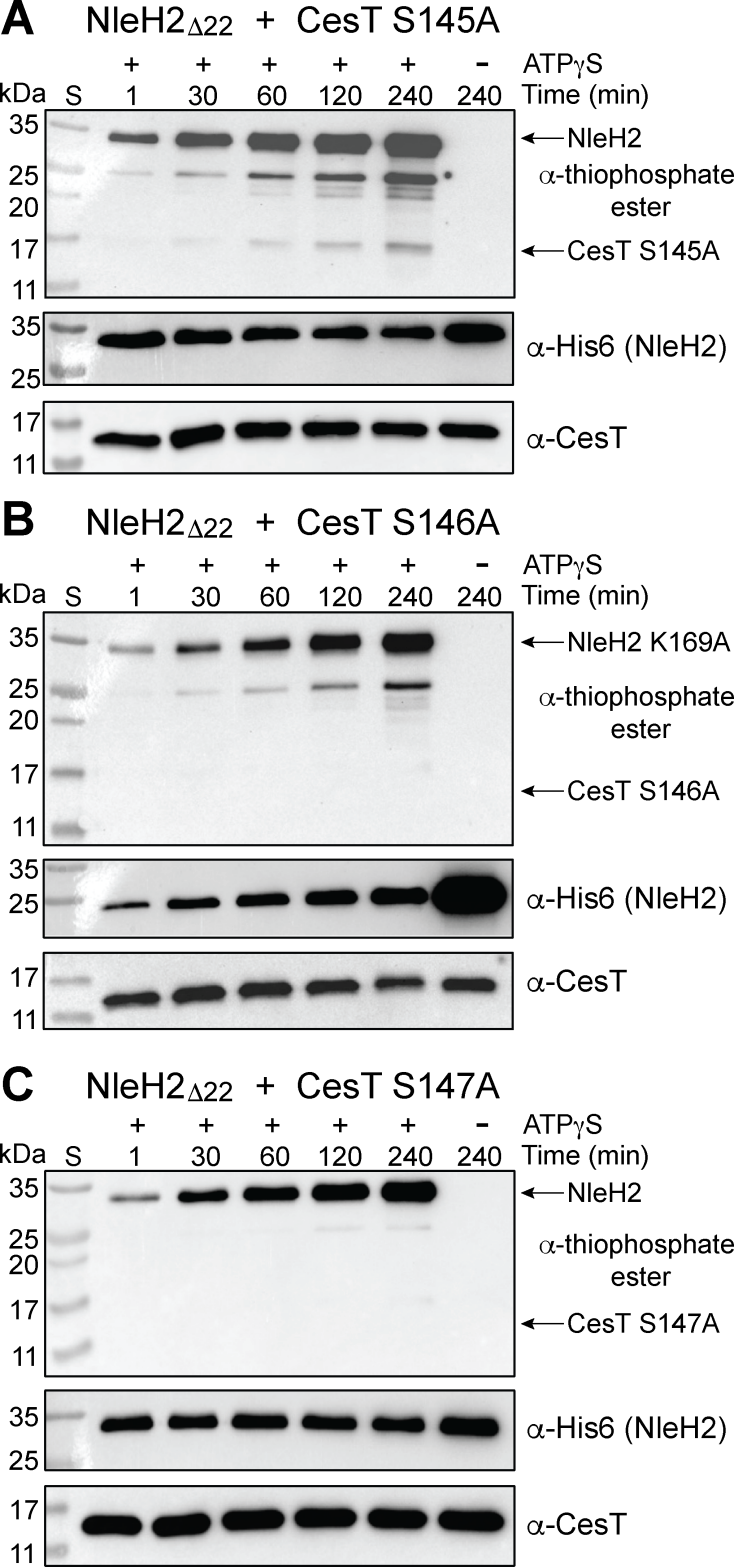
NleH2_Δ22_ shows limited to no phosphorylation of the CesT S146A and S147A phosphomutants. Purified His6-NleH2_Δ22_ co-expressed and purified with CesT phosphovariants (**A**) S145A, (**B**) S146A, and (**C**) S147A (4 µM) were incubated with 1 mM ATPγS and 2 mM MgCl_2_. Samples were taken at different time points and alkylated by incubating with 2.5 mM PNBM for 1 h. Samples were then run on SDS-PAGE and analyzed using α-thiophosphate ester, α-His6, and α-CesT western blot. The blots shown are representative of ≥2 independent experiments.

To experimentally test our structural model predictions, we cloned the S145A, S146A, and S147A CesT phospho-variants for co-expression with NleH2_Δ22_. Each complex was then expressed, purified, and assayed for phosphotransfer as previously described. Under these conditions, CesT S145A showed γ-phosphotransfer signal in the α-thiophosphate ester blot similar to WT CesT (Fig. 3A and 5A). This suggests that CesT S145 may not be a specific phosphosite for NleH2. However, CesT S146A and CesT S147A showed little to no γ-phosphotransfer signal in the α-thiophosphate ester blot compared to WT and S145A CesT (Fig. 3A, 5B and C). Together, these data suggest that CesT S146 and S147 are the primary phosphosites for NleH2_Δ22_ kinase activity.

### CesT S146E, S147A, and S147E phosphomutants show impaired ability to pull down with CsrA

**Fig 6:**
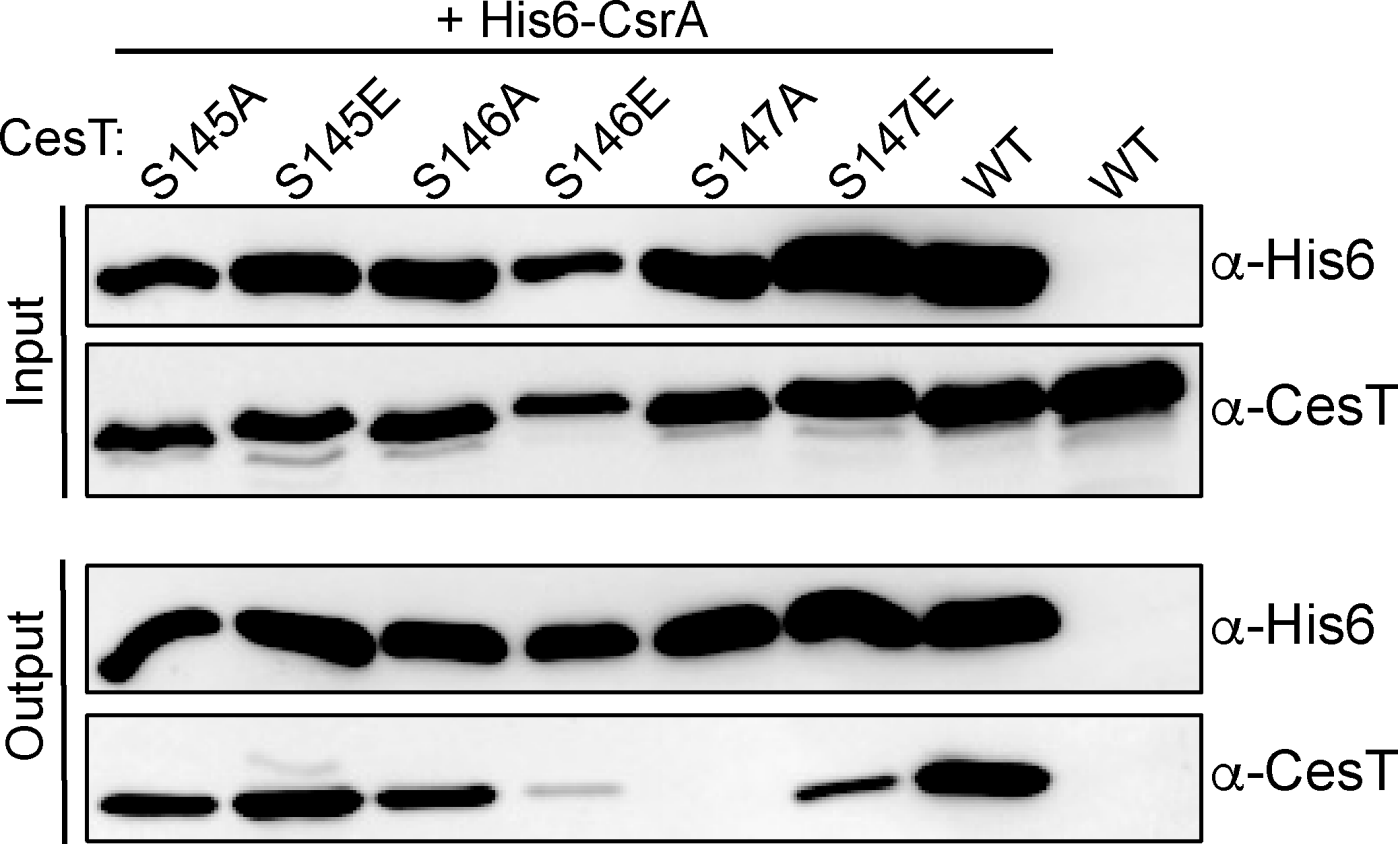
CesT S146E, S147E, and S147A phosphomutants show impaired ability to interact with His6-CsrA. Cells co-expressing His6-CsrA with untagged CesT S145A, S145E, S146A, S146E, S147A, or S147E were expressed and isolated using a Ni-NTA affinity pulldown. The soluble lysate fraction (input) and the pull-down elution fraction (output) of each sample was analyzed using α-His6 and α-CesT western blot analysis. Untagged WT CesT without co-expression with His6-CsrA was used as a negative control. The blots shown are representative of ≥2 independent experiments.

Now that we have identified S146 and S147 as the CesT phosphosites for NleH2, we wanted to determine the downstream biological consequence(s) for phospho-regulation of CesT. We hypothesized that phospho-CesT may be impaired for binding to interacting proteins, such as a T3SS effector or CsrA. To date, there have not been significant data suggesting that the C-terminus of CesT plays a major role in effector interactions. For example, CesT 1–138 has been shown to directly interact with Tir and NleH2 when co-expressed and purified (15, 21), and an affinity column loaded with CesT 1–145 was still capable of capturing many EPEC T3SS effectors from EPEC culture supernatants (20). On the other hand, the C-terminus of CesT has been shown to directly contribute to CesT self-dimerization and CsrA complex formation (18). More specifically, CesT Y152 was shown to play a critical role in CsrA binding, as a Y152A mutant completely abolished the interaction (18). Considering the proximity of the S145, S146, and S147 to CesT Y152, these sites could also play a role in CsrA interaction. Thus, we wanted to test if mutations to CesT S145, S146, or S147 altered CesT interaction with CsrA. To do this, we cloned His6-CsrA into a co-expression plasmid with CesT, and the individual S145, S146, and S147 variants, then conducted a co-expression and pull-down assay using Ni-NTA agarose resin. Specifically, we tested the CesT nonphosphorylatable mutants S145A, S146A, and S147A, and the phosphomimetic mutants S145E, S146E, and S147E. Cellular lysates expressing His6-CsrA and CesT (or variant thereof) were applied to Ni-NTA agarose resin, extensively washed, eluted with imidazole, and analyzed by SDS-PAGE and western blot. Our results showed that His6-CsrA and CesT (or a variant thereof) were expressed and present in all samples tested (Fig. 6). As for the elution fractions, complete pulldown of CesT was observed for the S145A and S145E variants, being comparable to CesT WT. This suggests that this site is not important for interaction and supports our earlier work showing it is not a target for NleH2 phosphorylation. In contrast, we saw reduced CesT in the elution samples for the S146E and S147E mutants and no pull-down ability of CesT S147A. This suggests two things: first, that S147 is important for complex formation with CsrA, and second, that the phosphorylation of S146 and S147 likely impairs the interaction between CesT and CsrA. The latter is because a glutamic acid side chain mimics that of a phospho-serine and would introduce a charge and steric hindrance to interacting residues. To further support this, we conducted structural modeling of phospho-S146 and -S147 (pS146 and pS147) CesT (Fig. S2 and S3). The models predict that CesT pS146 would not interact with CsrA E54 as seen in the WT CesT structure but shifts its interaction to CsrA Q58 (Fig. S2). Moreover, CesT Y152 still interacts with CsrA H43 and R44, albeit differently in this model (Fig. S2). The addition of the phosphate group to S146 seems to also reduce interactions of CesT with the C-terminal extension of the other CesT protomer in the complex (Fig. S2). This disruption would only impact the self-association of the C-termini and not the core CesT dimerization interface, which is supported by previous native PAGE analysis on a CesT 1–145 truncation that readily forms dimers in solution (20). Structural modeling of CesT pS147 shows even more disruptive changes when modeled with CsrA. In this model, the addition of the phosphate group causes CesT S147 to be pushed out and facing CsrA instead of the other CesT protomer, while also pushing S146 to an inward-facing position (Fig. S3). A close-up snapshot shows that interactions of CesT Y152 and S146 with the α1 helix of CsrA have been lost, plus the S147 interaction with the α2 helix of another CesT molecule is also disrupted from the phosphate group and causes drastic re-arrangement of the α4 helix (Fig. S3). Together, these results are congruent to our ATPγS phosphorylation assays showing S146 and S147 as the primary targets for NleH2 kinase activity, which support a model where phosphorylation of S146 and/or S147 influences the ability of CsrA to form a stable complex with CesT.

### EPEC Δ*nleH1* Δ*nleH2* shows increased levels of NleA translation and translocation

**Fig 7:**
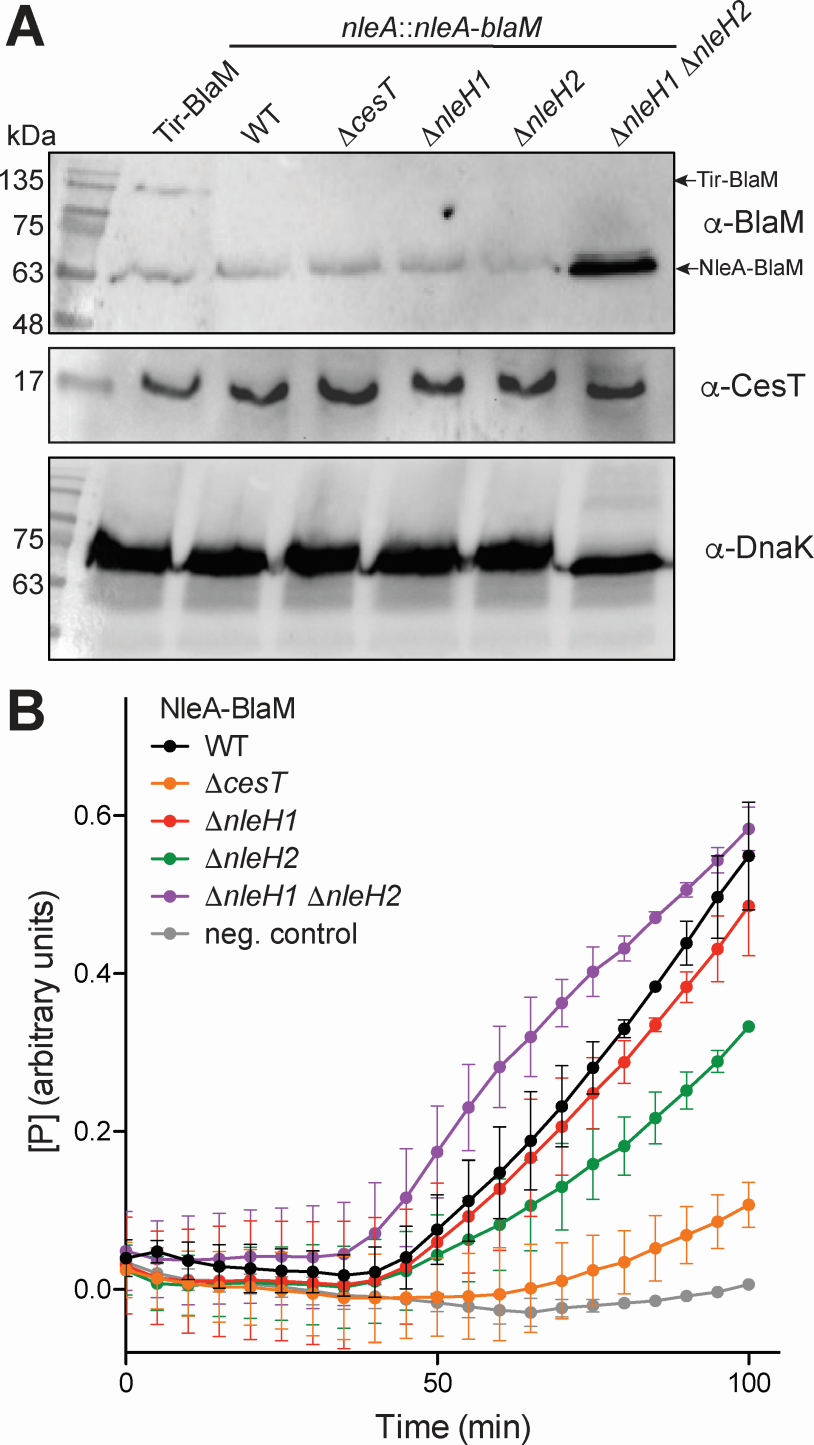
EPEC Δ*nleH1* Δ*nleH2* shows increased translation and translocation of NleA-BlaM under T3SS-inducing conditions. (**A**) EPEC WT expressing Tir-BlaM and NleA-BlaM, or EPEC ΔcesT, ΔnleH1, ΔnleH2, and ΔnleH1 ΔnleH2 expressing NleA-BlaM were grown in T3SS-inducing M9 optimized medium. The bacteria were harvested, lysed, separated via SDS-PAGE, and analyzed by western blot using α-BlaM, α-CesT, and α-DnaK. The blots shown are representative of ≥2 independent experiments. (**B**) EPEC WT (black line), ΔcesT (orange line), ΔnleH1 (red line), ΔnleH2 (green line), and ΔnleH1 ΔnleH2 (purple line) carrying the NleA-BlaM fusion were preactivated in T3SS-inducing medium before infection of HeLa cells loaded with CCF2/AM dye. EPEC WT without NleA-BlaM (gray line) was used as a negative control. The plots show the mean value of each time point, with error bars representing the standard deviation from two biological replicates. [P] is the ratio of blue:green fluorescence from the CCF2/AM dye displayed in arbitrary units (au).

As the phospho-mimetic mutants of CesT (S146E and S147E) showed reduced ability to interact with CsrA, we hypothesized that EPEC lacking the ability to phosphorylate CesT at these phosphosites would also alter CsrA repression of the *nleA* mRNA transcript. To test this, we integrated a *nleA-blaM* fusion into the chromosome of EPEC using a suicide plasmid that integrates at the native *nleA* locus. This approach has been used successfully in the past for monitoring effector translocation dynamics (12). Moreover, we created the *nleA-blaM* fusions in EPEC with Δ*cesT*, Δ*nleH1, nleH2*, and Δ*nleH1 nleH2* genetic backgrounds. Next, EPEC WT, Δ*cesT,* Δ*nleH1,* Δ*nleH2,* and Δ*nleH1* Δ*nleH2* carrying the NleA-BlaM fusion, and EPEC WT carrying the Tir-BlaM fusion, were grown in T3SS-inducing conditions and the whole-cell lysates were extracted and analyzed using α-BlaM western blot to monitor NleA-BlaM translation. EPEC expressing Tir-BlaM was used as a positive control for validation of the assay and antibody specificity. The upper band at about 130 kDa corresponds to Tir-BlaM, whereas the second band at 63 kDa is a non-specific band, which is also visible in all samples (Fig. 7A). EPEC WT, Δ*cesT,* Δ*nleH1,* and Δ*nleH2* strains showed no detectable signals outside of the non-specific band that would correspond to NleA-BlaM. However, there was a notable increase in NleA-BlaM translation in the EPEC Δ*nleH1* Δ*nleH2* genetic background, as seen in the strong signal just below the 63 kDa non-specific band (Fig. 7A). DnaK was used as a loading control to show equal amounts of sample applied during the analysis, and we also probed for CesT to ensure that the NleA-BlaM levels were not the result of increased or decreased CesT in the knockout strains (Fig. 7A).

To further corroborate our translational analysis, we also used the same strains to infect and monitor the real-time translocation of NleA-BlaM into host cells loaded with the β-lactamase-cleavable fluorescent dye CCF2/AM. If NleA-BlaM is present within HeLa cells, the CCF2 dye is cleaved, which produces an increased ratio of blue to green fluorescence due to loss of fluorescence resonance energy transfer (FRET) within the CCF2 molecule. Thus, an increase of the blue:green fluorescence ratio (denoted as [P]) within HeLa cells during infection represents increasing translocation of NleA-BlaM into host cells. The curves in Fig. 7B represent the mean of two biological replicates collected every 5 min with error bars representing the standard deviation. As shown in Fig. 7B, a chromosomal NleA-BlaM fusion in EPEC shows an increase of the blue:green fluorescence ratio starting at about 40 min after infection and increased steadily until completion of the assay at 100 min. Conversely, EPEC Δ*cesT* showed minimal increase in the blue:green fluorescence ratio from 70 to 100 min post-infection, and EPEC WT without NleA-BlaM showed no increase throughout the assay (Fig. 7B). This result is in line with previously reported translocation dynamics of NleA in these strains (12). Next, we tested the EPEC Δ*nleH1,* Δ*nleH2*, and Δ*nleH1* Δ*nleH2* strains expressing NleA-BlaM. EPEC Δ*nleH1* and Δ*nleH2* both showed increasing levels of the blue:green fluorescence ratio from about 45 min after infection until completion of the assay, with Δ*nleH1* and Δ*nleH2* tracking similar or at a slightly slower rate compared to WT, respectively (Fig. 7B). Interestingly, the EPEC Δ*nleH1* Δ*nleH2* strain showed a slight burst of NleA-BlaM translocation between 40 and 80 min before tapering off in comparison to WT (Fig. 7B). Together, these data suggest that the individual *nleH1* and *nleH2* knockouts do not show translational differences, as they would display redundant activity on CesT. Whereas the double *nleH1 nleH2* mutant lacks the ability to serine-phosphorylate CesT, thus allowing for an increase in the CesT population that could bind to CsrA and allow for increased NleA translation and translocation during host attachment and infection.

## DISCUSSION

CesT is an atypical class IA T3SS chaperone that plays a large part in governing effector secretion hierarchy by the T3SS in EPEC, EHEC, and *C. rodentium*. This regulation occurs in part through interactions with the chaperone binding domain(s) of an effector and/or post-translational modification of the CesT C-terminus. In particular, it was found that the tandem tyrosine residues Y152 and Y153 on CesT were phosphorylated (17), and mutating these residues to nonphosphorylatable variants showed reduced effector secretion and translocation of NleA into host cells by EPEC, as well as reduced host colonization through a murine model of infection using *C. rodentium* (19). More recently, it was shown that NleH2 in complex with CesT Y153F displayed decreased ADP generation (as a proxy for NleH2 phosphorylation) compared to WT CesT, and it was proposed that NleH2 was responsible for CesT tyrosine phosphorylation at the Y153 phosphosite (21). However, their interpretation relied on the production of ADP that would not distinguish between the loss of a Tyr phospho-acceptor (Y153F) and altered CesT-NleH2 binding. Thus, we investigated if NleH2—and by proxy NleH1—could alter CesT tyrosine phosphorylation levels. Our results did not show a reduction or difference between His6-CesT WT, Y152F, and Y153F in Δ*nleH1,* Δ*nleH2,* and the Δ*nleH1* Δ*nleH2* knockout strains, suggesting that NleH1 and NleH2 are likely not involved in tyrosine phosphorylation of CesT. However, we could reconcile the observation from Yadav et al. if there was a reduced ability of the CesT C-terminus (harboring the Y153F mutation) to bind to the NleH2 kinase domain active site. This is well supported by our structural modeling of NleH2 bound to a CesT C-terminal peptide where CesT Y153 contributes to the CesT-NleH2_KD_ interface (Fig. 4C). The Y153F mutation would have a high probability of reducing affinity for binding to the active site cleft, and in turn, decreasing ATPase activity of NleH2, providing an alternative explanation for the reduced ADP signal observed. Thus, the more plausible explanation of NleH1 and NleH2 phosphorylation specificity would be that of a serine or threonine residue in line with its activity as a serine kinase on host targets (25–27). This is congruent with our phosbind LC/MS-MS analysis (Table S4) and MS data from Yadav et al. identifying the S145/S146/S147 phosphosite(s) (21), and is supported by our ATPγS kinase assays showing markedly reduced γ-phosphate transfer levels using CesT S146A and S147A variants (Fig. 5).

As NleH2 is a highly translocated effector that is dependent on CesT, this suggests that phosphorylation would occur when CesT is binding to NleH2 in the cytosol prior to loading the effector into the T3SS sorting platform (12). The timing of this post-translational modification makes sense biologically, as there is a large pool of effectors in the bacterial cytosol that would be bound to CesT prior to T3SS-mediated host attachment (11, 12, 15). Moreover, CesT phosphorylation by NleH1 and/or NleH2 at S146 or S147 would not alter their interaction, or CesT interaction with other effectors in general. Thus, we posited that phosphorylation of CesT S146 and/or S147 by NleH1 and/or NleH2 could be a switch to impair or enhance CesT binding to proteins other than T3SS effectors. Indeed, this is what we observed for CesT interaction with CsrA. Ye et al. showed CsrA interacts with the C-terminus of CesT at Y152, where a Y152A mutant abolishes CsrA interaction (18). Similarly, we showed that CsrA was unable to pulldown a CesT S147A variant (Fig. 6). However, neither of these results provided insight for how phosphorylation alters the CsrA-CesT interface. Getz et al. investigated this for CesT Y152 by running molecular simulations of CsrA with CesT containing a phospho-Y152 (pY152) residue (28). They found that CsrA has a contiguous binding pocket that could mediate tripartite H-bonding coordination of the pTyr phosphate group, in comparison to a single H-bond formed from the Tyr hydroxyl group (28). This is also where CsrA binds the canonical GGA motif of mRNA targets (29–31), suggesting phosphorylation of CesT Y152 increases competition for this site (enhances binding to CsrA). This is not the case for CesT S146 or S147, as we observed that CsrA was impaired for pull-down of the CesT S146E and S147E variants (Fig. 6).

Overall, these results suggest a model where NleH1- and/or NleH2-dependent phosphorylation of CesT S146 and/or S147 negatively impacts CsrA-CesT interaction by repelling CsrA and/or changing the domain-swapped dimeric conformation of CesT that facilitates CsrA interaction. In either scenario, this would influence the translation of NleA as CesT binding to CsrA leads to de-repression of the *nleA* mRNA transcript (16). Indeed, monitoring the translation of NleA in EPEC Δ*nleH1* Δ*nleH2* during T3SS-inducing conditions showed markedly increased translation of NleA-BlaM (Fig. 7A). Furthermore, this also resulted in an increase in NleA-BlaM translocation into host cells during HeLa cell infection with EPEC Δ*nleH1* Δ*nleH2* compared to WT (Fig. 7B). This could be explained through an increased CesT population that is not phosphorylated at S146 or S147, allowing for more efficient antagonization of CsrA. This is congruent with Ramu et al. where they found that the phosphomimetic CesT mutants (S146E and S147E) had the lowest total protein secretion levels, with notable reduction in NleA, in an EPEC Δ*sepD* Δ*cesT* strain complemented *in trans* (20).

Together, this collection of studies allows us to posit a model for CesT regulation, where phosphorylation produces CesT subpopulations that mechanistically target different processes. In this scenario, CesT could exist in four defined states: (i) unphosphorylated, (ii) phosphorylated at S146 and/or S147, (iii) phosphorylated at Y152, and (iv) phosphorylated at Y153. In (i), the unphosphorylated CesT population would be the initial state and could be available for binding to either an effector or CsrA. Evidence suggests that CesT primarily binds Tir initially as co-translation of the LEE5 transcript is a known mechanism to balance the Tir-CesT ratio during early infection (32). This is because having excess free CesT early during T3SS activation would negatively impair EPEC colonization due to the premature sequestration of CsrA (32). For (ii), CesT pS146 and pS147 would push the equilibrium away from CsrA interaction that would limit NleA translation and translocation, which is supported by prior work (20), and could allow for lower translocated effectors to get recognized by CesT. Conversely, for (iii), CesT pY152 would be biased toward CsrA interaction, as evident by prior modeling and NleA translocation studies (19, 28). Lastly, (iv), CesT pY153 would be biased toward effector interaction, which is supported by increased effector translocation rates seen in a Y153F variant (19). By having these defined subpopulations of CesT, it could allow for continued secretion of effectors during infection, while sequestering CsrA at the appropriate concentration. Having some free CsrA might be required for the post-transcriptional regulation in other important processes such as central carbon metabolism, bacterial motility, and biofilm formation, needed by EPEC and EHEC during host attachment and infection (33–37). Likewise, after the initial release of Tir into the host cell, the bacteria would not benefit from all of the CesT molecules binding to CsrA, as that would drastically reduce the secretion of middle to lower translocated effectors, while increasing NleA translocation levels. These CesT phosphorylation states likely co-exist and are dynamic such that a balanced equilibrium between CesT-CsrA can be maintained during infection. The largest outstanding question then is why would EPEC and EHEC take such intricate regulatory steps to control NleA translation and translocation into host cells? It is known that NleA is an important effector for infection, as it targets various host cellular pathways such as inhibiting host vesicle transport and inflammasome activity, among others (38–42). It is possible that the timing of NleA delivery is important during the time course of an EPEC and/or EHEC infection, and future directions should look at changes within the host from modulating NleA translocation dynamics. Regardless, the biological implications of modulating CesT function presented herein further show the importance in phospho-regulation of effector secretion hierarchy and the T3SS in general.

## MATERIALS AND METHODS

### Strains and cloning

Bacterial strains, plasmids, and oligonucleotide primers used in this study are described in Tables S1 and S2. Phusion polymerase (Thermo Fisher Scientific) was used for all regular PCR reactions, oligonucleotide primers were synthesized by MilliporeSigma, restriction enzymes and T4 ligase were from Thermo Fisher, and site-directed mutagenesis (SDM) was performed by encoding the mutation in the primer using inverse or around-the-horn PCR with Q5 polymerase (NEB) and parental template digest by DpnI. In order to set up a series of expression plasmids with compatible restriction sites for ease of cloning, we first constructed a modified version of pCOLADuet-1 that introduced a tobacco etch virus (TEV) cleavage recognition site after the N-terminal histidine tag and before the BamHI site in the first multiple cloning site (MCS). This was done by encoding the TEV recognition sequence within the reverse primer for insertion using restriction-based cloning with ApaI and BamHI. This first MCS of pCOLADuet-TEV was then excised and inserted into pET28a using NcoI and HindIII to produce pET28-TEV. Next, we used SDM to remove the internal BamHI site from pFLAG-CTC (pFLAG-CTCb), then amplified the pCOLADuet-TEV first MCS using inverse PCR for insertion using the NdeI and XhoI restriction sites into pFLAG-CTCb to create pFLAG-CTC-His6-TEV. CesT WT and the Y152F, Y153F, and Y152F Y153F mutants were then cloned into pET28-TEV and/or pFLAG-CTC-His6-TEV using inverse PCR and the BamHI/HindIII restriction sites. NleH2_KD_ (residues 140–303) was cloned into pET28-TEV using inverse PCR and the BamHI/SalI restriction sites. NleH1_Δ19_ (residues 20–293), NleH2_Δ22_ (residues 23–303), and CsrA were cloned into pCOLADuet with CesT in MCS2 using inverse PCR and the BamHI/SalI, BamHI/SalI, and BamHI/HindIII restriction sites, respectively. All plasmids were verified by sequencing (TCAG, The Hospital for Sick Children).

### Construction of chromosomal deletions in EPEC

Genetic knockouts of EPEC Δ*nleH1*, Δ*nleH2,* and Δ*nleH1* Δ*nleH2* were generated using the lambda red recombinase method (43). Primers containing a 48-nucleotide homology region of *nleH1* or *nleH2*, flanked by a 20-nucleotide priming site, were used to amplify linear PCR products using the pKD3 or pKD4 plasmid templates, respectively. In-frame marked mutants of EPEC replacing *nleH1* residues 17–278 or *nleH2* residues 17–288, with a chloramphenicol acetyltransferase (cat) or kanamycin resistance (kan) cassette, were constructed using one-step λ-red inactivation with pKD46 and the transformed linear PCR products (43). The antibiotic cassettes were then removed using plasmid pFLP2 and sucrose selection (44). The EPEC Δ*nleH1* Δ*nleH2* strain was made in the same manner but using the EPEC Δ*nleH1* strain with pKD46 transformed with the linear *nleH2-kan* PCR product. All deletion strains were verified by sequencing (TCAG).

### Antibodies and western blotting

Protein fractions were mixed in equal volume with 2× SDS-PAGE loading dye (62.5 mM Tris-HCl, pH 6.8, 1% [wt/vol] SDS, 10% glycerol, 2% [vol/vol] 2-mercaptoethanol, 0.001% [wt/vol] bromophenol blue) and heated for protein denaturation at 90°C for 5–10 min. Samples were then centrifuged at 15,000 *g* for 30 s before separation by SDS-PAGE. Blue Elf prestained protein ladder (FroggaBio) was also loaded onto each gel for molecular weight estimation. The wet-tank transfer method was used for western blotting using nitrocellulose membrane (Bio-Rad). Transferred membranes were blocked for 1 h with Tris-buffered saline with 0.05% (vol/vol) Tween 20 (TBST) containing either 3% (wt/vol) bovine serum albumin (Bioshop) or 5% (wt/vol) skimmed milk (Bio-Rad). Membranes were washed with TBST and rocked overnight at 4°C with primary antibodies (Table S3). Membranes were taken out of primary antibodies, washed with TBST five times for 5 min each, and incubated with peroxidase-conjugated secondary antibodies (Table S3) for 1 h at room temperature with rocking. Membranes were taken out of the secondary antibody and washed with TBST five times for 5 min each before imaging using the Clarity Western (Bio-Rad) or PicoPlus (Thermo Fisher) ECL substrates and a ChemiDoc touch (Bio-Rad). All antibodies were commercially available except for α-CesT. Polyclonal CesT antibodies were raised in rabbits by Biomatik (Kitchener, ON, CAN) by supplying them with highly pure recombinant CesT without the histidine tag expressed and purified from *E. coli* BL21-CodonPlus (DE3) with pET28-TEV-CesT.

### EPEC CesT tyrosine phosphorylation assays

pFLAG-CTC-TEV-His6-CesT WT, Y152F, and Y153F were transformed into EPEC WT, Δ*nleH1*, Δ*nleH2,* and Δ*nleH1* Δ*nleH2* genetic backgrounds. All strains were grown overnight in lysogeny broth (LB) containing 150 µg/mL of carbenicillin at 37°C with shaking. Overnight cultures were sub-cultured 1:50 into pre-warmed M9 optimized media (3.4 mM Na_2_HPO_4_, 2.2 mM KH_2_PO_4_, 0.9 mM NaCl, 0.9 mM NH_4_Cl, 4% wt/vol glucose, 2% wt/vol casamino acids, 5 mM MgSO_4_, 10 mM NaHCO_3_) and grown standing at 37°C with 5% CO_2_. After 2 h, 0.1 mM of isopropyl β-D-1-thiogalactopyranoside (IPTG) was added to each flask and grown until OD_600_ reached 0.8–1.0. Samples were centrifuged at 4,000 *g* for 10 min, the supernatant was discarded, and the resulting cell pellet was resuspended with Ni-NTA lysis buffer (50 mM Tris-HCl, pH 7.5, 300 mM NaCl, 10 mM imidazole) and lysed via sonication for 2 min total, 20 s on, 60 s off, at 15% amplitude on ice. Lysed samples were then centrifuged at 20,000 *g* for 20 min. The supernatant was collected and applied to a column pre-equilibrated with 0.5 mL Ni-NTA agarose slurry. The Ni-NTA agarose column was washed with 5 mL Ni-NTA wash 1 buffer (20 mM Tris-HCl, pH 7.5, 300 mM NaCl, 10 mM imidazole) and 5 mL wash 2 buffer (20 mM Tris-HCl pH 7.5, 300 mM NaCl, 20 mM imidazole) before elution with 1 mL of elution buffer (20 mM Tris-HCl, pH 7.5, 300 mM NaCl, 250 mM imidazole). Elution samples were collected for western blot analysis using α-phosphotyrosine and α-His6 primary antibodies, and goat α-mouse peroxidase-conjugated secondary antibody (see Table S3). Western blots were analyzed for densitometric analysis using the Bio-Rad Imaging Software using the ratio of phosphotyrosine to His6-CesT protein levels.

### LC-MS/MS analysis

EPEC WT transformed with pFLAG-CTC-TEV-His6-CesT or pFLAG-CTC-TEV-His6-CesT Y152F Y153F were grown and the His6-CesT proteins isolated, exactly as indicated above for the EPEC CesT Tyrosine Phosphorylation Assay. Elution samples of His6-CesT and His6-CesT Y152F Y153F were mixed with 2× SDS-PAGE loading dye and separated on a 12% phosbind reagent acrylamide (APExBIO) gel made based on the Wako Laboratory Chemicals user manual for Zn^2+^ Phos-tag gel. The Phos-binding SDS-PAGE gel was run at 4°C, 30 mA using 3-(N-morpholino)propane sulfonic acid (MOPS) running buffer for ~4 h or until the 25 kDa marker of the protein ladder reached the bottom of the gel cassette. The gel was then stained with R250 Coomassie blue stain for 10 min, then destained (10% [vol/vol] methanol and 10% [vol/vol] acetic acid in ddH_2_O) overnight. The destained gel was then washed with water to remove excess destain. Phosbind SDS-PAGE separated bands were gel-extracted using a fresh blade and sent for trypsinization, LC-MS/MS, and PTM analysis at the SPARC Biocentre (The Hospital for Sick Children). Results were analyzed using Protein Scaffold software (Table S4).

### Structural modeling

Structural modeling of NleH2_KD_ was completed using PDB 4LRK in PyMOL with the addition of ATP as predicted using the AlphaFold 3 server. Structural modeling of NleH2 with ATP and a CesT C-terminal peptide (containing residues 138–156) was completed using AlphaFold 3 and analyzed in PyMOL. Structural modeling predictions of CesT and the pS146 and pS147 variants in complex with CsrA were completed using PDB 5Z38 and/or the AlphaFold 3 server, with analysis in PyMOL.

### Protein expression and purification for kinase assays

*E. coli* BL21-CodonPlus (DE3) cells were transformed with individual or co-expression plasmids (see Table S1) and grown overnight in LB containing 50 µg/mL of kanamycin at 37°C with shaking. Overnight cultures were sub-cultured 1:50 into 2 L of LB with 50 µg/mL of kanamycin and grown until OD_600_ = 0.4–0.6. Cultures were cooled to 18°C for 1 h before being induced with 1 mM IPTG and left shaking at 18°C overnight. Cultures were harvested by centrifugation at 5,000 *g* for 15 min, with the supernatant being decanted and the cell pellet collected. Cells were resuspended in 25 mL of lysis buffer (50 mM Tris, pH 7.5, 300 mM NaCl, 20 mM imidazole, 5% glycerol) per 1 L of culture pellet and lysed via sonication for two rounds of 2.5 min total, 15 s on, 60 s off, 45% amplitude, letting cool for 10 min in between rounds. Cell lysates were centrifuged at 30,000 *g* for 30 min, and the supernatants were added to a column containing 3 mL of Ni-NTA agarose pre-equilibrated with lysis buffer. The Ni-NTA-bound proteins were then washed with seven column volumes (CVs) of lysis buffer and 3 CVs wash buffer (20 mM Tris, pH 7.5, 1 M NaCl, 70 mM imidazole, 0.05% Tween 20, 5% glycerol) before eluting with elution buffer (20 mM Tris, pH 7.5, 300 mM NaCl, 250 mM imidazole). Ni-NTA affinity chromatography samples were stained and analyzed using SDS-PAGE as previously described. The Ni-NTA elution sample was then buffer-exchanged using a PD10 desalting column (Cytiva) into low-salt buffer (20 mM Tris, pH 7.5, 20 mM NaCl) and loaded onto a pre-equilibrated 1 mL HiTrap Q XL anion-exchange column (Cytiva). The column was washed with 20 CVs of low-salt buffer, then eluted with 40 CVs using a linear gradient from 0 to 100% with high-salt buffer (20 mM Tris, pH 7.5, 500 mM NaCl). Fractions were collected, analyzed by SDS-PAGE, and pure fractions were pooled and concentrated for injection into a HiLoad 16/60 S200 size-exclusion column (Cytiva) pre-equilibrated with size-exclusion buffer (20 mM Tris-HCl, pH 7.5, 150 mM NaCl). Column fractions were collected, analyzed by SDS-PAGE, and fractions with the purest protein were pooled, concentrated using an Amicon 10 kDa cutoff ultrafiltration device (Millipore Sigma), aliquoted, and frozen at −80°C.

### ATPγS kinase assays

Purified proteins or complexes were used to conduct ATPγS kinase assays. A stock solution containing 4 µM of the protein(s) of interest, 1 µM of ATPγS (Abcam), 2 µM of MgCl_2_, and size-exclusion buffer to bring the total volume to 200 µL was prepared. The stock solution was incubated for 4 h at 30°C, with sample aliquots removed at 1, 30, 60, 120, and 240 min after addition of ATPγS. Sample aliquots (40 µL) were immediately transferred to a 1.5 mL microfuge tube containing 2.4 mM *p*-nitrobenzyl mesylate (PNBM, Abcam) and alkylated for 1 h at 30°C. Reactions were stopped after alkylation using an equal volume of 2× SDS-PAGE loading dye and heating the sample for 5 min at 90°C. Assay samples were used for western blot analysis using α-thiophosphate ester, α-His6, and α-CesT primary antibodies and goat α-mouse or α-rabbit peroxidase-conjugated secondary antibodies.

### CsrA-CesT pull-down experiments

*E. coli* BL21-CodonPlus (DE3) cells were transformed with co-expression plasmids for His6-CsrA and CesT, or variants thereof (see Table S1), and grown overnight in LB containing 50 µg/mL of kanamycin at 37°C with shaking. Overnight cultures were sub-cultured 1:50 into 50 mL of LB with 50 µg/mL of kanamycin and grown until OD_600_ = 0.4–0.6. Cultures were cooled to 18°C for 1 h before being induced with 1 mM IPTG and left shaking at 18°C overnight. Cultures were harvested by centrifugation at 4,000 *g* for 10 min, with the supernatant being decanted and the cell pellet collected. Cells were resuspended in 3 mL of Ni-NTA lysis buffer and lysed via sonication for 2 min total, 20 s on, 60 s off, 15% amplitude. Cell lysates were centrifuged at 14,000 *g* for 20 min, and the supernatants were added to a column containing 0.5 mL of Ni-NTA agarose slurry pre-equilibrated with lysis buffer. Ni-NTA-bound proteins were then washed with 5 mL Ni-NTA wash 1 buffer and 5 mL Ni-NTA wash 2 buffer before being eluted with elution buffer. Soluble lysate and elution fraction samples were mixed with equal parts of 2× SDS-PAGE loading dye and analyzed by SDS-PAGE and western blotting using α-His6 and α-CesT primary antibodies, and goat α-mouse or α-rabbit peroxidase-conjugated secondary antibodies, respectively (Table S3).

### NleA-BlaM translation and translocation assays

Donor plasmid pHG3768 (*nleA::nleA-blaM*) or pCX442 (PLEE5-*tir::tir-blaM*) in *E. coli* SM10 λ-pir was used to conjugate the suicide plasmid into the recipient strains of EPEC WT, Δ*cesT*, Δ*nleH1,* Δ*nleH2,* and Δ*nleH1* Δ*nleH2*. Positive clones were isolated using LB agar plates with tetracycline and streptomycin (LB-tet-strep) at 12 and 50 µg/mL, respectively. Single colonies were then re-streaked on fresh LB-tet-strep agar plates and used to inoculate LB-tet-strep media with overnight growth at 37°C with shaking. For monitoring NleA-BlaM translation, the overnight cultures were sub-cultured 1:50 into 50 mL of M9 optimized media (3.4 mM Na_2_HPO_4_, 2.2 mM KH_2_PO_4_, 0.9 mM NaCl, 0.9 mM NH_4_Cl, 4% [wt/vol] glucose, 2% [wt/vol] casamino acids, 5 mM MgSO_4_, 10 mM NaHCO_3_) and grown standing at 37°C with 5% CO_2_ until OD_600_ = 0.8–1.0. Cultures were centrifuged at 4,000 *g* for 10 min, the supernatant was discarded, and the resulting pellets were resuspended and normalized with 2× SDS-PAGE loading dye, boiled at 90°C for 10 min, separated by SDS-PAGE, and analyzed by western blot analysis using α-BlaM, α-CesT, and α-DnaK primary antibodies, and goat α-mouse or rabbit secondary antibody (Table S3).

Host cell translocation assays were completed as described previously (12, 13, 45). Briefly, overnight cultures of Tir- or NleA-BlaM fusion strains (as detailed above) were sub-cultured 1:100 into pre-equilibrated fluorobrite DMEM (Gibco) and grown standing at 37°C with 5% CO_2_ for ~2.5–3 h (OD_600_ = 0.25–0.35). Bacterial cultures were then normalized to OD_600_ = 0.25 using Fluorobrite DMEM (Gibco). One day before the translocation assay, HeLa cells were seeded in 96-well black with clear bottom tissue culture plates at 5 × 10^4^ cells in DMEM + 10% fetal bovine serum (FBS) (MilliporeSigma). On the day of infection and 1.5 h into the EPEC sub-culture, HeLa cells were loaded at room temperature with CCF2/AM dye using the LiveBLAzer FRET-B/G loading kit (Thermo Fisher) as per the manufacturer’s directions using DMEM + 10% FBS for 75 min in the dark, then for an additional 15 min at 37°C with 5% CO_2_. HeLa cells were then washed four to six times with phosphate-buffered saline and infected with bacterial cultures at an MOI of ~1,000:1. Immediately after infection, the plate was sealed and placed into a Cytation 5 plate reader set to 37°C, and the infection was allowed to proceed for 100 min reading every 5 min at an excitation of 409 nm and emission at 450 nm and 520 nm (all 20 nm bandwidths). Data were collected with Gen5 software, processed in Excel, and analyzed in Prism. The blue:green fluorescence ratio [P] was calculated as P = (B_in_ – B_Bk_) / (G_in_ – G_Bk_), where B_in_ and B_Bk_ are the measured product (blue) fluorescence at 450 nm of infected cells with BlaM fusions and WT EPEC (background signal negative control) loaded with CCF2, respectively; and G_in_ and G_Bk_ are the measured substrate (green) fluorescence at 520 nm of infected cells with BlaM fusions loaded with CCF2 and infected cells without CCF2, respectively. The infection assay results are from two biological replicates conducted in technical duplicates.

## SUPPLEMENTAL MATERIAL

Supplemental material iai.00027-26-s0001.pdf Tables S1 to S4; Fig. S1 to S3.

ASM does not own the copyrights to Supplemental Material that may be linked to, or accessed through, an article. The authors have granted ASM a non-exclusive, world-wide license to publish the Supplemental Material files. Please contact the corresponding author directly for reuse.
