# Supplementary material for: Serine phosphorylation of CesT by the type III secretion system effectors NleH1 and NleH2 regulates antagonization of CsrA in enteropathogenic Escherichia coli
Source: Infect Immun. 2026 Feb 26;94(4):e00027-26. doi: 10.1128/iai.00027-26 (PMC13081723; doi:10.1128/iai.00027-26)
Supplement: Supplemental material — Tables S1 to S4; Fig. S1 to S3. [file iai.00027-26-s0001.pdf]

Serine phosphorylation of CesT by the type III secretion system effectors NleH1 and NleH2 regulates antagonization of CsrA in enteropathogenic *Escherichia coli*

Esther Tang[a], Angeline C. Beltran[a], Senthuran Mahendradeva[a], Abiali A. Badani[a], and Dustin J. Little[a]#

[a] Department of Chemistry and Biology, Toronto Metropolitan University, Toronto, ON M5B 2K3, Canada

# Correspondence can be sent to Dustin J. Little, djlittle@torontomu.ca

Running title: Serine phosphorylation of CesT by NleH effectors

Table S1. Strains and plasmids used in this study.

Strain or plasmid	Description or characteristics	Source/reference
**Strains**		
EPEC E2348/69	Enteropathogenic *E. coli* isolate O127:H6 str. E2348/69 (Str^r)	1
EPEC Δ*cesT*	EPEC E2348/69 Δ*cesT*, CesT deficient strain	2
EPEC Δ*nleH1*	EPEC E2348/69 Δ*nleH1*, NleH1 deficient strain	This study
EPEC Δ*nleH2*	EPEC E2348/69 Δ*nleH2*, NleH2 deficient strain	This study
EPEC Δ*nleH1* Δ*nleH2*	EPEC E2348/69 Δ*nleH1* Δ*nleH2,* NleH1 and NleH2 deficient strain	This study
TOP10	*E. coli* cloning strain: F^- *mcr*A Δ(*mrr-hsd*RMS-*mcr*BC) ϕ80*lac*ZΔM15 Δ*lac*X74 *rec*A1 *ara*D139 Δ(*ara-leu*) 7697 *gal*U *gal*K *rps*L (Str^r) *end*A1 *nup*G λ-	Invitrogen
BL21 CodonPlus (DE3)	*E. coli* expression strain: F^- *omp*T *hsd*S(r_B^- m_B^-) *dcm*$^+$ Tet^r *gal* λ(DE3) *end*A [*arg*U *pro*L Cam^r]	Stratagene
DH5α λ-pir	*E. coli* cloning strain: F⁻ *endA1 glnV44 thi-1 recA1 relA1 gyrA96 deoR nupG purB20* φ80d*lacZ*ΔM15 Δ(*lacZYA-argF*)U169, hsdR17($r_K^-m_K^+$), λpir^+	Lab stock
SM10 λ-pir	*E. coli* donor strain: *thi-1*, *thr*, *leu*, *tonA*, *lacY*, *supE*, *recA*::RP4-2-Tc::Mu, λpir^+, (Kan^R)	Lab stock
**Plasmids**		
pET28a	Expression vector	Novagen
pET28-TEV	Expression vector, TEV-cleavable N-His6	This study
pCOLADuet-1	Co-expression vector	Novagen
pCOLADuet-TEV	Co-expression vector, TEV-cleavable N-His6	This study
pFLAG-CTC	Expression vector	MilliporeSigma
pFLAG-CTCb	Expression vector, internal BamHI site removed	This Study
pFLAG-CTC-His6-TEV	Expression vector, TEV-cleavable N-His6	This Study
pET28-TEV-CesT	CesT expression plasmid, TEV-cleavable N-His6 tag	This study
pET28-TEV-$NleH2_{KD}$	NleH2 Kinase Domain (140-303) expression plasmid, TEV-cleavable N-His6 tag	This study
pET28-TEV-$NleH2_{KD}$ K169A	NleH2 Kinase Domain (140-303) K169A expression plasmid, TEV-cleavable N-His6 tag	This study
pCOLADuet-$NleH1^{20\text{-}293}$-CesT	Co-expression plasmid for N-His6 tagged NleH1 20-293 and CesT	This study
pCOLADuet-$NleH2^{23\text{-}303}$-CesT	Co-expression plasmid for N-His6 tagged NleH2 23-303 and CesT	This study
pCOLADuet-$NleH2^{23\text{-}303}$ K169A-CesT	Co-expression plasmid for N-His6 tagged NleH2 23-303 K169A and CesT	This study
pCOLADuet-$NleH2^{23\text{-}303}$-CesT S145A	Co-expression plasmid for N-His6 tagged NleH2 23-303 and CesT S145A	This study
pCOLADuet-$NleH2^{23\text{-}303}$-CesT S146A	Co-expression plasmid for N-His6 tagged NleH2 23-303 and CesT S146A	This study
pCOLADuet-$NleH2^{23\text{-}303}$-CesT S147A	Co-expression plasmid for N-His6 tagged NleH2 23-303 and CesT S147A	This study
pCOLADuet-MCS2-CesT	Co-expression plasmid for untagged CesT in the second MCS	3
pCOLADuet-CsrA-CesT	Co-expression plasmid for N-His6 tagged CsrA and CesT	This study
pCOLADuet-CsrA-CesT S145A	Co-expression plasmid for N-His6 tagged CsrA and CesT S145A	This study
pCOLADuet-CsrA-CesT S145E	Co-expression plasmid for N-His6 tagged CsrA and CesT S145E	This study
pCOLADuet-CsrA-CesT S146A	Co-expression plasmid for N-His6 tagged CsrA and CesT S146A	This study
pCOLADuet-CsrA-CesT S146E	Co-expression plasmid for N-His6 tagged CsrA and CesT S146E	This study
pCOLADuet-CsrA-CesT S147A	Co-expression plasmid for N-His6 tagged CsrA and CesT S147A	This study

pCOLADuet-CsrA-CesT S147E	Co-expression plasmid for N-His6 tagged CsrA and CesT S147E	This study
pKD3	λ-red chloramphenicol template plasmid, pir-dependent	4
pKD4	λ-red kanamycin template plasmid, pir-dependent	4
pKD46	λ-red recombinase expression plasmid	4
pFLP2	FLP recombinase expression plasmid, *sacB*$^+$	5
pFLAG-CTC-TEV-His6-CesT	CesT expression plasmid, TEV-cleavable N-His6 tag	This study
pFLAG-CTC-TEV-His6-CesT Y152F	CesT Y152F expression plasmid, TEV-cleavable N-His6 tag	This study
pFLAG-CTC-TEV-His6-CesT Y153F	CesT Y153F expression plasmid, TEV-cleavable N-His6 tag	This study
pFLAG-CTC-TEV-His6-CesT Y152F Y153F	CesT Y152F Y153F expression plasmid, TEV-cleavable N-His6 tag	This study
pCX442	Suicide plasmid for generation of chromosomal *tir-blaM* fusion at the native locus	6
pHG3768	Suicide plasmid for generation of chromosomal nleA-*blaM* fusion at the native locus	7

Table S2. List of primers used in this study.

Primer	Gene	Direction	Residue	Destination	Sequence
DL708	pFLAG-CTC	Fwd	nt 5186	pFLAG-CTC	GTCCGGCGTAGAGGCTCCGGGCTTATCGACTG
DL709	pFLAG-CTC	Rev	nt 5185	pFLAG-CTC	GCATCGTGGCCGGCATCACCGGCGCCAC
DL145	pCOLADuet	Fwd	nt 3021	pCOLADuet	GGAATTGGGCCCGCTAACAGCGCG
DL146	pCOLADuet	Rev	nt 106	pCOLADuet	GGAATTGGATCCGACTGGAAATACAGGTTCTCCTGGCTGTGGTGATG
DL631	pCOLADuet-TEV	Fwd	nt 238	pET28a, pFLAG-CTCb	GGAATTCATATGGGCAGCAGCCATCAC
DL632	pCOLADuet-TEV	Rev	nt 158	pET28a, pFLAG-CTCb	GGAATTCTCGAGTGCGGCCGCAAG
DL504	*nleH1*	Fwd	20	pCOLADuet	GGAATTGGATCCGCTGACTTCTCCTGAC
DL458	*nleH1*	Rev	293	pCOLADuet	GGAATTGTCGACCTAAATTTTACTTAATAC
DL930	*nleH2*	Fwd	23	pCOLADuet	GGAATTGGATCCTGATAATCGTGTTTTA
DL840	*nleH2*	Fwd	140	pET28-TEV	GGAATTGGATCCGAATAAATCACCCGTG
DL421	*nleH2*	Rev	303	pCOLADuet, pET28-TEV	GGAATTGTCGACTTATATCTTACTTAATACTACAC
DL922	*nleH2*	Fwd	K169A	pCOLADuet, pET28-TEV	CAACAAAAGTGTTGGCGATGTTTACTATATCTCAAAGCC
DL923	*nleH2*	Rev	K169A	pCOLADuet, pET28-TEV	GATATAGTAAACATCGCCAACACTTTTGTTGTATCTTCC
DL946	*nleH1*	Fwd	1	Δ*nleH1*	ATGCTATCACCATCTTCTGTAAATTTGGGGTGTTCATGGAATTCTTTAGTGTAGGCTGGAGCTGCTTC
DL947	*nleH1*	Rev	293	Δ*nleH1*	CTAAATTTTACTTAATACCACACTAATAAGATCTTGCTTTCCTCCATGCATATGAATATCCTCCTTAG
DL948	*nleH1*	Fwd	nt -60	Screening primer	CATGTCATGGTGATGTTTGTTAAGAAAGTAAAG
DL949	*nleH1*	Rev	nt +101	Screening primer	CAGCGGCATTGTCCATTTCTGTGAC

DL950	*nleH2*	Fwd	1	Δ*nleH2*, Δ*nleH1* Δ*nleH2*	ATGTTATCGCCCTCTTCTATAAATTTGGG ATGTTCATGGAATTCTTTAGTGTAGGCTG GAGCTGCTTC
DL951	*nleH2*	Rev	303	Δ*nleH2*, Δ*nleH1* Δ*nleH2*	TTATATCTTACTTAATACTACACTAATAAG ATCCAGCTTTCCTCCGTGCATATGAATAT CCTCCTTAG
DL952	*nleH2*	Fwd	nt -57	Screening primer	GTACGGGGGATGTCTGTTAGGAATAATTA G
DL953	*nleH2*	Rev	nt +91	Screening primer	GCTGAAGAACCACTTGTTGGTAACA
DL622	*cesT*	Fwd	2	pET28-TEV, pFLAG-CTC-TEV-His6	TAGAGGAAATTGCCTCAAGCGATAATAAA CATTATTAC
DL36	*cesT*	Rev	156	pET28-TEV, pFLAG-CTC-TEV-His6	GAGCCTCGAGTTATCTTCCGGCGTA
DL307	*cesT*	Rev	156 + Y152F	pFLAG-CTC-TEV-His6	GGAATTCTCGAGTTATCTTCCGGCGTAAA AATGTTTATTATCGC
DL308	*cesT*	Rev	156 + Y153F	pFLAG-CTC-TEV-His6	GGAATTCTCGAGTTATCTTCCGGCGAAAT AATGTTTATTATCGC
DL309	*cesT*	Rev	156 + Y152F Y153F	pFLAG-CTC-TEV-His6	GGAATTCTCGAGTTATCTTCCGGCGAAAA AATGTTTATTATCGCT
DL955	*cesT*	Fwd	S145A	pCOLADuet	TAGAGGAAATTGCCTCAAGCGATAATAAA CATTATTAC
DL956	*cesT*	Rev	S145A	pCOLADuet	GTTTATTATCGCTTGAGGCAATTTCCTCT ATTTTC
DL957	*cesT*	Fwd	S145E	pCOLADuet	TAGAGGAAATTGAGTCAAGCGATAATAAA CATTATTAC
DL958	*cesT*	Rev	S145E	pCOLADuet	GTTTATTATCGCTTGACTCAATTTCCTCTA TTTTC
DL959	*cesT*	Fwd	S146A	pCOLADuet	GAGGAAATTAGCGCCAGCGATAATAAAC ATTATTACGC
DL960	*cesT*	Rev	S146A	pCOLADuet	TAATGTTTATTATCGCTCGCGCTAATTTCC TCTATTTTC
DL961	*cesT*	Fwd	S146E	pCOLADuet	GAGGAAATTAGCGAAAGCGATAATAAACA TTATTACGC
DL962	*cesT*	Rev	S146E	pCOLADuet	TAATGTTTATTATCGCTTTCGCTAATTTCC TCTATTTTC
DL963	*cesT*	Fwd	S147A	pCOLADuet	GGAAATTAGCTCAGCCGATAATAAACATT ATTACGCCG
DL964	*cesT*	Rev	S147A	pCOLADuet	TAATGTTTATTATCGGCTGAGCTAATTTCC TCTATTTTC
DL965	*cesT*	Fwd	S147E	pCOLADuet	GGAAATTAGCTCAGAGGATAATAAACATT ATTACGCCG
DL966	*cesT*	Rev	S147E	pCOLADuet	TAATGTTTATTATCCTCTGAGCTAATTTCC TCTATTTTC
DL304	*csrA*	Fwd	1	pCOLADuet-CesT (MCS2)	GGAATTGGATCCGATGCTGATTCTGAC
DL305	*csrA*	Rev	61	pCOLADuet-CesT (MCS2)	GGAATTAAGCTTTTAGTAACTGGACTGCT GG

Table S3. List of antibodies used in this study.

Type	Name	Company	Dilution
Mouse monoclonal IgG2b, primary	anti-phosphotyrosine 4G10 platinum	MilliporeSigma (05-1050)	1:2000, in TBST
Mouse monoclonal IgG2a, primary	anti-His6	MilliporeSigma (H1029)	1:5000, in TBST
Rabbit recombinant monoclonal IgG, primary	anti-thiophosphate ester	Abcam (ab92570)	1:5000, in TBST
Rabbit polyclonal, primary	anti-CesT	Custom, Biomatik (AB014241)	1:50000, in TBST
Mouse monoclonal IgG1, primary	anti-TEM1 (BlaM)	Abcam (ab122151)	1:2000, in TBST + 5% BSA
Mouse monoclonal IgG1, primary	anti-DnaK	Enzo Life Sciences (ADI-SPA-880-J)	1:5000, in TBST
Goat polyclonal IgG, secondary	anti-mouse peroxidase-conjugated	Jackson Immuno (115-035-003)	1:10000, in TBST + 1% skimmed milk or BSA
Goat polyclonal IgG, secondary	anti-rabbit peroxidase-conjugated	Jackson Immuno (111-035-003)	1:10000, in TBST + 1% skimmed milk or BSA

Table S4. LC-MS/MS and Scaffold PTM analysis of phosbind acrylamide SDS-PAGE separated CesT and CesT Y152F Y153F samples.

**Site**	**Modification**	**Best Ascore**	**Localization Probability**	**CesT WT top band**	**CesT WT bottom band**	**CesT Y152F Y153F top band**	**CesT Y152F Y153F bottom band**
S5	Phospho	68.13	1.00	0	1	0	0
S20	Phospho	60.18	1.00	0	2	0	0
Y124	Phospho	101.08	1.00	0	1	3	1
T133	Phospho	53.83	1.00	1	1	8	0
S145	Phospho	23.20<br>50.64	0.99<br>1.00	4	4	6	7
S147	Phospho	17.98	0.97	1	0	0	0

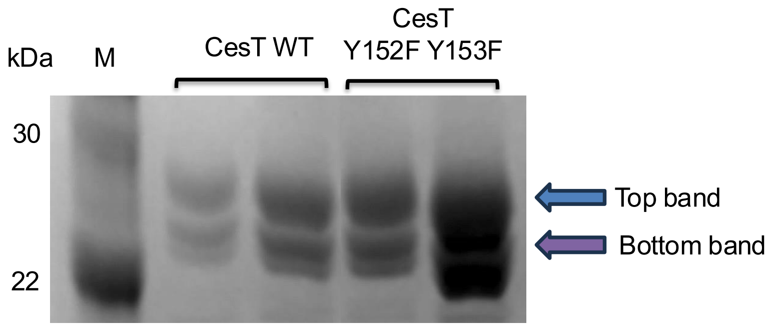

Figure S1. Phosbind acrylamide SDS-PAGE analysis of CesT and CesT Y152F Y153F.

His6-CesT WT and His6-CesT Y152F Y153F were expressed in EPEC under T3SS-inducing conditions with M9 optimized media, lysed, and isolated using Ni-NTA resin. Each sample was then run on an 11% phosbind acrylamide SDS-PAGE, stained with coomassie R250, and showed multiple phospho-CesT species. The top and bottom SDS-PAGE bands were excised, thoroughly destained, and sent for trypsinolysis followed by LC-MS/MS, and PTM analysis at SickKids SPARC Biocentre. M, molecular weight standards.

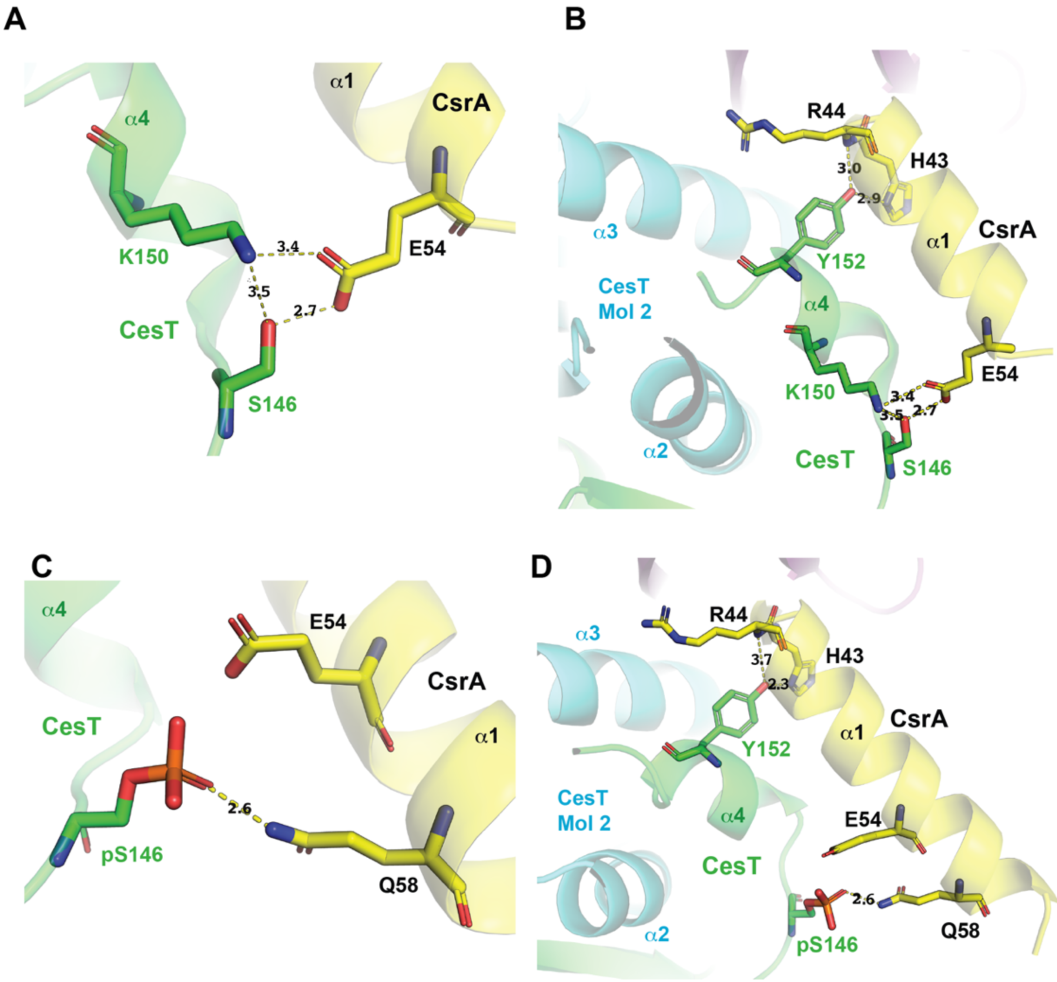

Figure S2. Structural modeling of CesT S146 and pS146 with CsrA.

Interactions of WT CesT in complex with CsrA shows (**A**) CesT S146 (green) interacting with CsrA E54 (yellow) and CesT K150 (green), and (**B**) CesT Y152 (green) interacting with CsrA H43 and R44 (yellow). Interactions of phospho-S146 (pS146) CesT in complex with CsrA shows (**C**) CesT pS146 (green) loses hydrogen bonding to CsrA residue E54 (yellow) and now interacts with CsrA Q58 (yellow), and (**D**) CesT Y152 (green) interacting with CsrA H43 and R44 (yellow) with altered hydrogen bonding. Hydrogen bonding distances (Å) are shown as yellow dashes. Structures were modelled using AlphaFold 3, visualized and aligned onto the PDB 5Z38 structure with PyMOL.

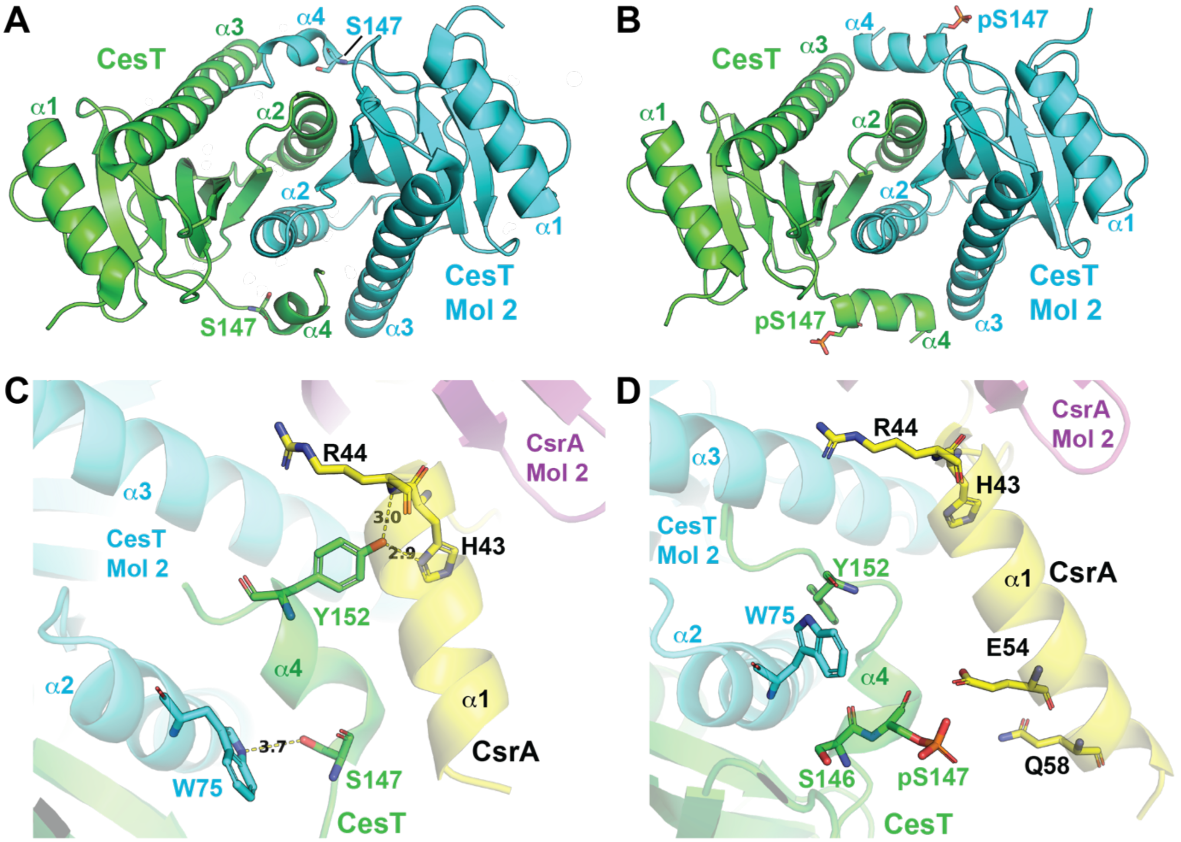

Figure S3. Structural modeling of CesT S147 and pS147 with CsrA.

Comparison of the CesT (**A**) WT and (**B**) pS147 structures shows an altered domain swapped dimeric conformation of α4. Interactions of WT CesT in complex with CsrA shows (**C**) CesT S147 (green) interacting with CesT W75 (cyan), and CesT Y152 (green) interacting with CsrA H43 and R44 (yellow). Interactions of phospho-S147 (pS147) CesT in complex with CsrA shows (**D**) CesT pS147 (green) loses hydrogen bonding to CesT W75 (cyan) and now flips out with no specific interactions. and CesT Y152 (green) flips away from CsrA H43 and R44 (yellow) with no hydrogen bonding interactions. Hydrogen bonding distances (Å) are shown as yellow dashes. Structures were modelled using AlphaFold 3, visualized and aligned onto the PDB 5Z38 structure with PyMOL.

References

(1) Levine, MyronM.; Nalin, DavidR.; Hornick, RichardB.; Bergquist, ErickJ.; Waterman, DanielH.; Young, CharlesR.; Sotman, S.; Rowe, B. ESCHERICHIA COLI STRAINS THAT CAUSE DIARRHŒA BUT DO NOT PRODUCE HEAT-LABILE OR HEAT-STABLE ENTEROTOXINS AND ARE NON-INVASIVE. *The Lancet* **1978**, *311* (8074), 1119–1122. https://doi.org/10.1016/S0140-6736(78)90299-4.

(2) Abe, A.; De Grado, M.; Pfuetzner, R. A.; Sánchez-SanMartín, C.; DeVinney, R.; Puente, J. L.; Strynadka, N. C. J.; Finlay, B. B. Enteropathogenic Escherichia Coli Translocated Intimin Receptor, Tir, Requires a Specific Chaperone for Stable Secretion. *Mol. Microbiol.* **1999**, *33* (6), 1162–1175. https://doi.org/10.1046/j.1365-2958.1999.01558.x.

(3) Little, D. J.; Coombes, B. K. Molecular Basis for CesT Recognition of Type III Secretion Effectors in Enteropathogenic Escherichia Coli. *PLOS Pathog.* **2018**, *14* (8), e1007224. https://doi.org/10.1371/journal.ppat.1007224.

(4) Datsenko, K. A.; Wanner, B. L. One-Step Inactivation of Chromosomal Genes in Escherichia Coli K-12 Using PCR Products. *Proc. Natl. Acad. Sci.* **2000**, *97* (12), 6640–6645. https://doi.org/10.1073/pnas.120163297.

(5) Hoang, T. T.; Karkhoff-Schweizer, R. R.; Kutchma, A. J.; Schweizer, H. P. A Broad-Host-Range Flp-*FRT* Recombination System for Site-Specific Excision of Chromosomally-Located DNA Sequences: Application for Isolation of Unmarked *Pseudomonas Aeruginosa* Mutants. *Gene* **1998**, *212* (1), 77–86. https://doi.org/10.1016/S0378-1119(98)00130-9.

(6) Mills, E.; Baruch, K.; Charpentier, X.; Kobi, S.; Rosenshine, I. Real-Time Analysis of Effector Translocation by the Type III Secretion System of Enteropathogenic Escherichia Coli. *Cell Host Microbe* **2008**, *3* (2), 104–113. https://doi.org/10.1016/j.chom.2007.11.007.

(7) Mills, E.; Baruch, K.; Aviv, G.; Nitzan, M.; Rosenshine, I. Dynamics of the Type III Secretion System Activity of Enteropathogenic Escherichia Coli. *mBio* **2013**, *4* (4), 10.1128/mbio.00303-13. https://doi.org/10.1128/mbio.00303-13.
